# Interactions between parental traits, environmental harshness and growth rate in determining telomere length in wild juvenile salmon

**DOI:** 10.1111/mec.13857

**Published:** 2016-10-14

**Authors:** D. McLennan, J. D. Armstrong, D. C. Stewart, S. Mckelvey, W. Boner, P. Monaghan, N. B. Metcalfe

**Affiliations:** ^1^ Institute of Biodiversity, Animal Health and Comparative MedicineUniversity of GlasgowGraham Kerr BuildingGlasgowG12 8QQUK; ^2^ Marine Scotland – ScienceFreshwater LaboratoryFaskallyPitlochryPH16 5LBUK; ^3^ Cromarty Firth Fishery TrustCKD GalbraithReay House17 Old Edinburgh RoadInvernessIV2 3HFUK

**Keywords:** environmental, growth, parental, Salmo, telomere

## Abstract

A larger body size confers many benefits, such as increased reproductive success, ability to evade predators and increased competitive ability and social status. However, individuals rarely maximize their growth rates, suggesting that this carries costs. One such cost could be faster attrition of the telomeres that cap the ends of eukaryotic chromosomes and play an important role in chromosome protection. A relatively short telomere length is indicative of poor biological state, including poorer tissue and organ performance, reduced potential longevity and increased disease susceptibility. Telomere loss during growth may also be accelerated by environmental factors, but these have rarely been subjected to experimental manipulation in the natural environment. Using a wild system involving experimental manipulations of juvenile Atlantic salmon *Salmo salar* in Scottish streams, we found that telomere length in juvenile fish was influenced by parental traits and by direct environmental effects. We found that faster‐growing fish had shorter telomeres and there was a greater cost (in terms of reduced telomere length) if the growth occurred in a harsher environment. We also found a positive association between offspring telomere length and the growth history of their fathers (but not mothers), represented by the number of years fathers had spent at sea. This suggests that there may be long‐term consequences of growth conditions and parental life history for individual longevity.

## Introduction

A larger body size has many benefits, such as increased reproductive success, ability to evade predators and increased competitive ability and social status (Blanckenhorn 2000; Dmitriew 2011). Attaining a large size requires either prolonged or faster growth. However, there is evidence that individuals rarely maximize their growth rates, suggesting that there are costs associated with rapid growth (Metcalfe & Monaghan 2003). It is becoming clearer that one such cost may be reduced longevity. Within species, even when comparing adults of the same size, those with faster growth rates earlier in life often exhibit faster senescence and/or shorter lifespans (Metcalfe & Monaghan 2003; Geiger *et al*. 2012). There are likely to be multiple underlying mechanisms linking growth rate and faster senescence. Several studies have found growth rate to correlate positively with levels of oxidative damage, both in the laboratory and in the field (Alonso‐Álvarez *et al*. 2007; Nussey *et al*. 2009; Carney Almroth *et al*. 2012), and oxidative damage levels are considered to be important influences on longevity and senescence (Halliwell & Gutteridge 2015). In addition, growth rate may alter trade‐offs between body growth and body maintenance (i.e. antioxidant production and protein repair). McCarthy *et al*. (1994) reported such a trade‐off in rainbow trout (*Oncorhynchus mykiss*), with faster‐growing individuals undergoing increased protein synthesis (growth) and reduced protein turnover (maintenance). The costs of a particular pace of growth may also, in turn, be partly dependent on environmental factors. For example, Stier *et al*. (2014) found that faster growth in coal tits *Periparus ater* was more costly (in terms of DNA oxidative damage) when they were reared under environmentally harsher conditions. Therefore, it may not only be the rate at which you grow, but also the environment in which you do so that has an effect on the costs and benefits of different growth trajectories.

Telomere length might be a good indicator of these costs. Telomeres cap the ends of eukaryotic chromosomes and play an important role in chromosome protection (for reviews, see Blackburn 1991; Campisi *et al*. 2001). Some telomere loss inevitably occurs at each cell division as a result of the ‘end replication problem’ (Chan & Blackburn 2004), so telomere dynamics are affected by the pattern and rate of cell division. In addition to this, telomere loss may also be accelerated by the effects of reactive oxygen species (ROS). Telomeric DNA is vulnerable to oxidative damage for a number of reasons, particularly its high G content (Haussmann & Marchetto 2010). While the enzyme telomerase is capable of restoring telomere length, telomerase expression is downregulated in many types of somatic cells, which is thought to be linked to tumour suppression (Collins 2006). A relatively short telomere length is indicative of poor biological state, for example reduced potential longevity and increased disease susceptibility (Haussmann *et al*. 2005; Ilmonen *et al*. 2008; Calado & Young 2009; Heidinger *et al*. 2012).

The determinants of variation in telomere length among and within species are still not fully understood. This is especially the case for animals living in the wild, since thus far telomere dynamics and their consequences have mostly been studied in stable laboratory conditions. An increasing number of studies have reported a heritable component to telomere length. These studies have mostly focussed on humans (for example Nordfjall *et al*. 2005; Njajou *et al*. 2007); however, other taxa have also been studied, including lizards (Olsson *et al*. 2011) and many bird species (Horn *et al*. 2011; Asghar *et al*. 2015; Reichert *et al*. 2015). Results to date are inconsistent as to whether the inheritance is strongest through the father or mother (Eisenberg 2014).

Changes in telomere length are also strongly influenced by environmental conditions. As mentioned above, telomere attrition is related to the dynamics of cell proliferation and a number of studies have reported a relationship between enhanced growth rate or body size and telomere loss (Fick *et al*. 2012; Herborn *et al*. 2014; Noguera *et al*. 2015; Pauliny *et al*. 2015). These studies are mainly correlational. However, using transgenic coho salmon *Oncorhynchus kisutch* with an artificially increased growth rate, Pauliny *et al*. (2015) found that these faster‐growing fish showed a faster telomere loss compared with maternal half‐sibs that grew at a rate more typical of wild fish. On the other hand, Naslund *et al*. (2015) found that wild brown trout *Salmo trutta* induced to undergo compensatory growth did not show increased telomere loss. There have also been suggestions that telomere length may help explain the previously mentioned relationship between growth rate and longevity (Stindl 2004; Fick *et al*. 2012). Growth rate and body size are both under the influence of various environmental factors (Metcalfe & Monaghan 2001). This is especially true for ectotherms, such as fish, where fluctuations in temperature can influence myogenic processes, morphological development, growth rate and metabolism, all of which can have permanent long‐term phenotypic effects (Johnston 2006; Jonsson & Jonsson 2011). Therefore, it is also possible that the environmentally conditioned plasticity of growth rate may also have some influence on telomere dynamics. Environmental stressors may also affect telomere length, most notably through their influence on the production of ROS (von Zglinicki 2002; Geiger *et al*. 2012; Kim & Velando 2015). A number of experimental studies have linked telomere dynamics to environmental stressors such as in utero stress (Haussmann *et al*. 2011; Marchetto *et al*. 2016), stress hormone administration and disturbance (Herborn *et al*. 2014), social position (Nettle *et al*. 2015) and social crowding (Kotrschal *et al*. 2007; Sohn *et al*. 2012). Therefore, if growth rate does have an effect on telomere dynamics, its impact may also in turn be dependent on environmental conditions.

To gain a better understanding of telomere length as a possible link between environmental conditions, growth rate and individual fitness, it is also important that we study these processes in animals living in the wild. Using free‐living animals to tease apart parental and environmental effects can prove challenging, due to the logistics of accessing and obtaining data for parents and offspring alike. However, by utilizing managed populations of wild Atlantic salmon (*Salmo salar*), it is possible to examine environmental effects on telomere dynamics, while simultaneously investigating potential parental effects. Atlantic salmon exhibit intrapopulation variation in their life histories (for reviews see Fleming 1996; Klemetsen *et al*. 2003). They migrate to sea and undergo a substantial increase in body mass during this time, remaining there for either one sea winter (1SW) or multiple sea winters (MSW) before returning to fresh water to reproduce. MSW fish are generally much larger than 1SW fish when they return to freshwater (Klemetsen *et al*. 2003). There is a correlation between female body size and average egg size (Fleming 1996), and therefore, MSW mothers, in general, produce significantly larger eggs than 1SW mothers. It is also possible for males to become precociously mature as parr, prior to sea migration, and thus at a much smaller body size than males that go to sea to complete their growth (Fleming 1996; Baum *et al*. 2004). Salmon will also vary in the number of years spent in fresh water prior to seaward migration, with faster‐growing individuals reaching the minimum size threshold required for sea migration earlier and thus migrating to sea at a younger age (Metcalfe & Thorpe 1990; Økland *et al*. 1993). Therefore, within a typical Atlantic salmon population, there are two distinct maternal reproductive life‐history variants with respect to time spent at sea (1SW and MSW) and three such variants in males (1SW, MSW and precocious parr) and within a given sea age, individuals will vary in the number of years spent in freshwater prior to migration. As the rate of offspring growth is highly dependent on water temperature (Jonsson *et al*. 2001), spatial variation in water temperatures within a river catchment will mean that the exact location in which an egg is laid will influence the growth rate, and hence potentially the telomere dynamics, of the resulting offspring.

We used this system to examine parental and environmental effects on telomere dynamics in Atlantic salmon. We placed eggs from controlled matings of wild Atlantic salmon in contrasting streams within the same river system to test two hypotheses: (i) that faster growth is associated with increased telomere loss and (ii) that the magnitude of telomere loss for a given growth rate is greater in harsher conditions. In addition, we were also able to simultaneously investigate the effect of parental life history and parental telomere length on offspring telomere length in the wild.

## Methods

The experiment was conducted at the River Blackwater, which is part of the larger River Conon catchment, northern Scotland (57° 60′N, 4°63′W) (Fig. S1, Supporting information). Fish movements in the River Conon have been highly impacted by the installation of hydroelectric schemes since the 1950s. In particular, this has prevented fish from reaching many of the traditional spawning areas in tributary streams. The Loch na Croic Atlantic salmon trap was constructed on the River Blackwater in the late 1950s to maintain an Atlantic salmon population in the upper regions of the River Conon (i.e. above the hydroelectric schemes) by artificial spawning. This is carried out by collecting returning wild adults in the trap (1790 individuals per year ± SD 163; based on data from 1965 to 2012), conducting random crosses and then planting out developing eggs (mixed at random) into tributaries throughout the upper region of the catchment. No other stocking takes place in the Conon system. As all parent fish are collected from the same trap and their eggs are mixed thoroughly before being spread among the streams in the catchment, there is no reason to expect any genetic spatial differentiation in the catchment that would complicate this study.

**Figure 1:**
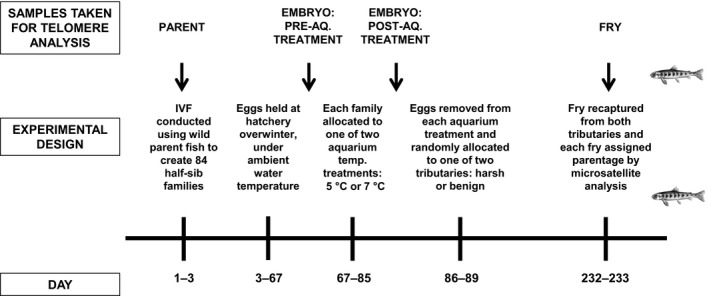
Outline of experimental design. Day 1 = 3 December 2013.

This set‐up allowed us to access large numbers of sexually mature wild fish, which were used to generate experimental families by in vitro fertilization (IVF). The experimental design (Fig. 1) was to first incubate the resulting eggs under contrasting aquarium temperatures (to examine the effect on telomeres of early embryonic conditions) and then plant the eggs in contrasting tributary streams: a lower altitude stream with moderate temperature and predator density and, in contrast, a harsher high‐altitude stream with a relatively colder temperature and greater predator density (as a consequence of differences in stocking with salmon eggs in previous years). In both streams, the mean temperature was below the optimum for growth (16 °C; Elliott and Hurley 1997) but it was furthest from the optimum in the harsher high‐altitude stream. The juvenile salmon were subsequently recaptured at a later date, and parental assignment was established by microsatellite analysis. This allowed the measurement of relative telomere length in parents and offspring (embryos and fry), all within a wild system.

The fish trap was first opened on 14th November 2013. The IVF matings for this experiment were all conducted between 3rd and 5th December, using predominantly fish captured the same day (and always within 1 week of capture). Unripe fish that could not be used in IVF crosses on the day of capture were held at the trap site in dark circular tanks (4 m diameter, 1.5 m deep), supplied directly with water from the adjacent river, until they had reached spawning condition. It is unknown whether fish of different life‐history strategies (i.e. 1SW and MSW) arrived at the loch downstream of the trap at different times. However, 1SW and MSW fish were both present by the time the trap was first opened, were treated in a similar way and were stripped over the same narrow time window. Sexually mature precocious male parr (i.e. small sneaker males, that had matured without going to sea) were captured daily between 3rd and 5th December, by electrofishing various tributaries of the River Blackwater. All captured parr were assessed for sexual maturity, and precocious males (indicated by the production of milt after abdominal squeezing) were retained for use that day in the IVF crosses.

In this experiment, we used a split‐brood IVF design. In each replication of the mating design, the eggs of two female fish (one 1SW and one MSW) were fertilized with sperm from three male fish (one 1SW, one MSW and one precocious male parr) to produce six half‐sib families that represented all possible parental crosses (Fig. S2, Supporting information). This design was repeated 14 times (using new fish each time) to produce 84 families in total. The life history of each of the anadromous parents was initially determined by size (MSW fish are generally much larger than 1SW fish (mean mass (g): MSW females = 3125 ± SE 101; 1SW females = 1368 ± SE 48; MSW males = 4199 ± SE 158; 1SW males = 1606 ± SE 123; male parr = 23 ± SE 2). This was subsequently confirmed by analysis of the scales taken from the flank of each fish (below the adipose fin) (Shearer 1992). The scale analysis also allowed us to retrospectively determine the number of years each parent fish had spent in fresh water prior to seaward migration, and confirmed that all parent fish were virgin spawners. There was not a strong correlation between the number of years parents had spent in FW and SW (Pearson *r* = 0.47, *P* < 0.001). Prior to the stripping of gametes, all parent fish were anaesthetized, using a 5% benzocaine solution. The two female fish in each mating group (1SW & MSW) were blotted dry and manually stripped of their clutch of eggs. The eggs were drained of ovarian fluid and then weighed (to 0.01 g) to obtain a clutch mass. For each female, a small sample of eggs (~15) was stored in 100% ethanol, for subsequent calculation of dry egg weight after overnight drying at 60 °C.

Each clutch was then divided into three approximately equal subsets. The three male fish (1SW, MSW and precocious parr) were blotted dry and manually stripped of sperm. The sperm of each male was divided into two, and each half was used to fertilize one subset of eggs from each of the two females. Each batch of eggs was left for several minutes to allow sufficient time for fertilization to occur. Afterwards, the eggs were washed in fresh water to remove any remaining sperm. Each parent fish was measured (fork length to 0.5 cm; body mass to 0.1 g). A small sample of tissue was taken from the adipose fin and stored in 100% ethanol for subsequent telomere analysis. All parent fish used in the crosses were then returned to the river, with adult salmon being returned just above the trap (so as not to be mixed up with previously untrapped individuals) and precocious parr returned to the section of stream from which they had been electrofished (which was not fished in subsequent days to avoid recapturing the same fish).

Eggs were transferred to the nearby SSE hatchery at Contin where they were held for 2 months in separate family groups under ambient water temperatures (3.90 ± SD 0.91 °C) until they had reached the more stable ‘eyed egg’ stage of development. On 05 February 2014, 10 eggs were sampled from each family for subsequent analysis of embryo telomere length, prior to the aquarium temperature treatments (see Fig. 1). A large and a small subsample of eggs (*n* = 500 and 30 eggs, respectively) was then counted from each family. The small subsamples were held as separate families in organza pouches to allow later collection of family‐specific data on telomere dynamics at the embryo stage, as the larger subsamples were to be subsequently mixed together. On 07 February 2014, each large subsample of eggs was allocated to one of two temperature treatments, depending on which family it belonged to: families with odd numbers (i.e. family 1 and family 3) were assigned to the 5 °C group and families with even numbers were assigned to the 7 °C group. This resulted in each of the 84 families being assigned randomly to only one temperature treatment, so that the aquarium temperature treatment of a fish could subsequently be known using genotyping to determine its family. For each treatment group, the large subsamples of eggs were mixed together to create two batches of pooled eggs (~21 000 eggs per batch). The two large batches of eggs (along with the small subsamples of each family in the organza pouches) were then transferred to the aquarium facilities at the University of Glasgow, where they were held in egg baskets in two separate dark tanks (100 × 100 × 40 cm). The small subsamples were allocated to their corresponding tank (families with odd numbers to the 5 °C group and families with even numbers to the 7 °C group) and remained in their pouches throughout the treatment. Both tanks were initially held at 5 °C, to match the ambient temperature at the hatchery facilities. The temperature in the 7 °C group was slowly raised to 7 °C over a 2‐day period. Eggs were exposed to these temperature treatments for 18 days. On February 25, the small subsamples of eggs were removed and stored in ethanol for subsequent analysis of embryo telomere length at the end of the aquarium temperature treatment period. The mixed eggs in the baskets of the two temperature treatment tanks were combined and mixed thoroughly to create a single large batch of ~42 000 eggs. This was then transferred back to the SSE hatchery at Contin, where the eggs were kept for several days under ambient water temperature conditions until being planted in the appropriate treatment streams.

Two tributaries (Upper Meig and Allt Goibhre) of the River Blackwater were selected to represent a harsh and a benign stream, respectively. While it is difficult to produce an absolute definition of ‘harsh’ and ‘benign’, we use these as relative terms to describe the conditions for growth in the two streams: the selected streams provided similar spawning substrate and water flow (Table S1, Supporting information), but while both showed a seasonal variation in temperature, the harsher high‐altitude stream (Upper Meig) was consistently colder and so further from the optimum temperature for growth (mean difference between streams = 0.72 ± SE 0.04 °C based on recordings logged every 4 h over the duration of the experiment; see Fig. S3, Supporting information). In addition, higher altitude tributaries often show reduced primary productivity, compared to relatively lower altitude tributaries of the same system (Elliott *et al*. 1998; Nislow *et al*. 2004). Both streams contained resident brown trout, and the Upper Meig also contained older juvenile Atlantic salmon that had been stocked as eggs in previous years. Both trout and older juvenile salmon predate Atlantic salmon fry (Henderson & Letcher 2003) and are hereafter combined and referred to as ‘predators’. Subsequent electrofishing showed that the harsher Upper Meig tributary had a ~3.6 × greater density of these predators than the Allt Goibhre tributary (Table S1, Supporting information). There was no natural salmon spawning in either stream due to migration barriers, so that the only salmon fry present were those introduced as part of the experiment.

The pooled batch of eggs was mixed again and then divided into two new batches (~21 000 eggs per batch), with each batch being assigned to one of the study streams. As a result, eggs from each of the aquarium temperature treatments were equally represented in each batch, and subsequently stream. The eggs were then planted in the benign and harsh streams on 26 February 2014 and 01 March 2014, respectively (see Supporting information for further details).

Both streams were then electrofished between 22 and 23 July 2014 to collect samples for telomere analysis and determine fry survival and growth rates. Beginning downstream of the treatment section, successive 10‐m stretches were measured out along the stream. Each section was sampled with a single electrofishing pass. All Atlantic salmon fry (which by definition were experimental fish) were euthanized with a 10% benzocaine solution and stored in 100% ethanol for subsequent body size measurements and tissue sampling in the laboratory. All nonexperimental fish (brown trout and older Atlantic salmon) were briefly anaesthetized, measured and then returned to the stream. For each 10‐m section, a number of habitat parameters were measured using the SFCC general electrofishing habitat survey (see Table S1, Supporting information). Consecutive sections were electrofished and analysed in this manner until ~400 experimental fry had been caught at each site. Fry density and predator density was calculated for each electrofishing site by dividing the total number of experimental fry or predatory salmonids (older brown trout and salmon) by the surface area of that site. All preserved experimental fry were weighed (0.001 g), and their fresh weight (mg) was estimated from the following equation: *M*_B1_ = 1.51*M*_B2_ + 70.69, where *M*_B1_ and *M*_B2_ are the fresh and ethanol preserved values, respectively (equation from Burton *et al*. 2013).

The caudal fin was removed from each fry and sent to Landcatch Natural Selection Ltd (Stirling, UK) for microsatellite analysis of parentage (see Supporting information). For the telomere analysis, DNA was extracted from all tissue types using the DNeasy Blood and Tissue Kit (Qiagen), following the manufacturers protocol, with a minor modification to the lysis step for each of the tissue types (see Supporting information). Telomere length was measured in all samples using the quantitative PCR method described by Cawthon (2002), which provides a relative measure of telomere length (RTL) and is calculated as a ratio (T/S) of telomere repeat copy number (T) to a control, single copy number (S) (here the glyceraldehyde‐3‐phosphate dehydrogenase (GAPDH) gene). Details of primers are given in the Supporting information. Telomere PCR conditions were 15 min at 95 °C followed by 27 cycles of 15 s at 95 °C, 30 s at 58 °C and 30 s at 72 °C. This was followed by the melt curve profile: temperature was slowly increased from 58 °C to 95 °C at a rate of 0.2 °C/s. GAPDH PCR conditions were 15 min at 95 °C followed by 40 cycles of 15 s at 95 °C, 30 s at 60 °C and 30 s at 72 °C. Again, this was followed by the melt curve profile (same as before). PCRs were performed on a Mx3005P qPCR system (Agilent).

The telomere (T) and single copy gene (S) assays were performed on separate 96‐well plates, with each sample run in triplicate for each assay. In addition to the samples, each plate also included a sixfold serial dilution of a reference sample (1.25–40 ng/well), a ‘golden reference’ sample and a nontarget control (NTC). The DNA for the serial dilution was a pool of 60 samples and included all life stages (adult, embryo and fry). The serial dilution was used to generate a standard curve and calculate assay efficiencies. The ‘golden standard’ was a pool of DNA from 20 samples, including all life stages, used as a reference sample. The NTC contained all reaction components apart from DNA and was included on each plate (in triplicate) to check for nonspecific binding and potential contamination between sample wells. Each reaction contained 12.5 μL 2x ABsolute Blue qPCR SYBR Green Mix low ROX (Fisher Scientific), forward and reverse primers and DNA (for wells containing sample, standards, gold reference) or water (for wells containing NTC) in a total volume of 25μL. Both T and S assays were performed using 10 ng of DNA (equivalent to 6 μL of the diluted samples). Primer concentrations were 500 nm for the telomere assay (Tel1b and Tel2b) and 200 nm for the GAPDH assay (salGAP8‐F and salGAP8‐R). The mean assay efficiencies for the telomere and GAPDH were 103.5% and 99.2%, respectively, and within the acceptable range (85–115%). The average intraplate variation of the *C*_t_ values was 1.15 for the telomere assay and 0.59 for the GAPDH assay, respectively. The average interpolate variation of the *C*_t_ values was 2.59 for the telomere assay and 1.86 for the GAPDH assay, respectively. qPCR raw data were analysed using qbase software for Windows (Hellemans *et al*. 2007). This controlled for differences in amplification efficiency between plates (assessed from the standard curve of each plate) and produces a relative quantity (RQ) for each gene of each sample. In addition, by including three inter‐run calibrators (the gold reference and two points from the standard curve), we corrected for further inter‐run variation. Finally, we used the software to normalize each telomere RQ by the GAPDH RQ for that sample. Therefore, for each sample, the qBASE software produced a calibrated normalized relative quantity (CNRQ). This is similar to the T/S ratio described by Cawthon (2002) but with greater control of interplate variation.

We measured/calculated the following four dependent variables: (A) fresh weight of each fry, calculated from the ethanol preserved weight values (*fry weight*); (B) the per cent of fry recaptured in July, calculated separately for each family in both streams (subsequently referred to as *fry survival rate*); (C) embryo relative telomere length, calculated as the mean for each family based on a pooled sample of 10 embryos (*embryo RTL*); and (D) fry relative telomere length, for each individual fry (*fry RTL*). All five of the dependent variables were assessed by linear mixed models (LME) that included maternal ID and paternal ID as random effects to control for nonindependence of siblings. We measured/calculated the following variables to be included as factors/covariates in analyses, where appropriate: the time point at which the embryo stage was sampled (i.e. whether before or after the aquarium temperature treatment, and subsequently referred to as *embryo time point*), the aquarium temperature treatment each family was kept under (*aquarium temperature*), the relative telomere length of mother and father (*maternal RTL* and *paternal RTL*), the number of years each parent spent in fresh water (*maternal FW age* and *paternal FW age*) and at sea (*maternal SW age* and *paternal SW age;* note that total age = FW age + SW age), average dry egg weight for each family (*egg weight*), which experimental stream a fry was reared in (*stream*), fry density for each electrofishing section within a stream (*fry density*) and predator density for each electrofishing section within a stream (*predator density*). Tables S2–S5 (Supporting information) describes the four full models, prior to model selection and simplification. The Akaike information criterion *(*AIC) was used during model fitting, and variables were only removed from a model if this resulted in a relative reduction in the AIC score. We used Pearson correlation coefficient matrices to assess potential collinearity between explanatory variables (with a cut‐off coefficient of 0.8). We also used Pearson correlation coefficient to assess the relationship between predator density and fry density. All statistical analyses were carried out using ibm spss 22 for Windows.

## Results

### Fry weight and fry density

As expected, fry weight (i.e. size achieved by late July, approximately 2 months after first feeding) differed between the two streams, being significantly greater in the more benign (and warmer) stream (Table 1A, Fig. S4, Supporting information). There was also a significant correlation between fry weight and fry density, with relatively smaller fry being present in areas of increased fry density (Table 1A, Fig. S5, Supporting information). Both fry weight and survival were positively related to initial egg weight (Table 1A and B, Figs S6 and S7, Supporting information). There was no significant correlation between predator density and fry density, either within streams or across streams (Pearson *r* < 0.30, *P* > 0.17).

**Table 1:** Summary of the four final linear mixed‐effect models explaining variation in: (A) fry weight (g), (B) fry survival, (C) embryo telomere length and (D) fry telomere length. See Supporting information for a full list of the main effects and interactions initially included in each model

Model	Explanatory variable	Numerator d.f.	Denominator d.f.	*F*	*P*
A	Stream	1	763.12	1199.89	<0.001
	Fry density	1	765.17	11.34	0.001
	Egg weight	1	50.08	20.44	<0.001
B	Egg weight	1	18.53	11.66	0.003
C	Embryo time point	1	100.84	22.37	<0.001
	Paternal RTL	1	41.58	4.38	0.042
D	Stream	1	733.96	2.52	0.113
	Fry weight	1	732.79	1.22	0.269
	Fry density	1	733.14	14.53	<0.001
	Predator density	1	736.60	14.98	<0.001
	Paternal SW age	3	106.11	3.859	0.012
	Fry weight × stream	1	728.81	13.02	<0.001
	Fry weight × fry density	1	731.55	8.51	0.004

### Embryo relative telomere length

**Figure 2:**
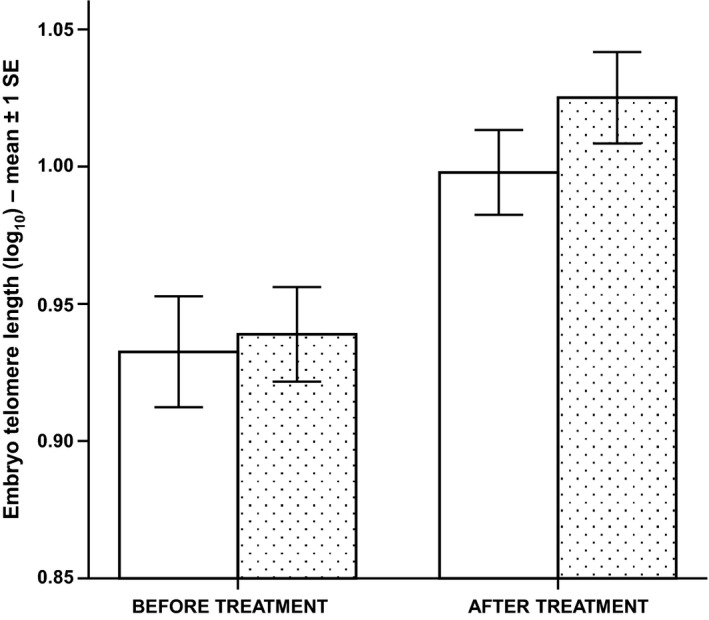
The mean relative embryo telomere length before and after the aquarium temperature treatment. Blank columns represent the 5 °C group, and the patterned columns represent the 7 °C group.

Embryo telomere length significantly increased during the 18 days of the aquarium temperature manipulation, with no difference between the two treatment groups (Table 1C, Fig. 2). Embryo telomere length was negatively correlated with paternal (but not maternal) telomere length, a pattern that was evident both before and after the temperature manipulation (Table 1C, Fig. S8, Supporting information).

### Fry relative telomere length

**Figure 3:**
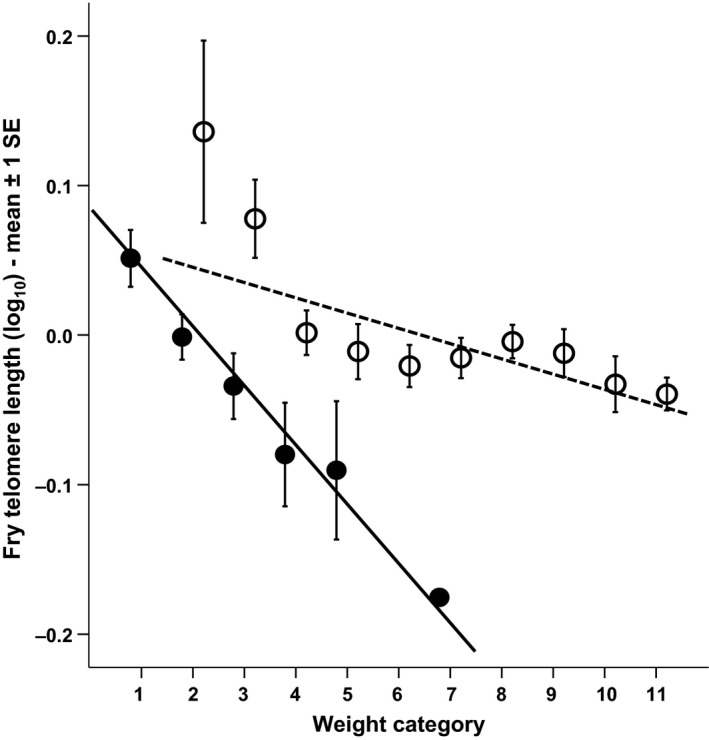
The relationship between fry weight (g) and fry telomere length. Closed circles = harsh stream and open circles = benign stream. Weight data grouped for ease of presentation (analysis based on original data); definition of weight groups (g): group 1 =< 0.40, group 2 = 0.41–0.50, group 3 = 0.51–0.60, group 4 = 0.61–0.70, group 5 = 0.71–0.80, group 6 = 0.81–0.90, group 7 = 0.91–1.00, group 8 = 1.01–1.10, group 9 = 1.11–1.20, group 10 = 1.21–1.30, group 11 => 1.31.

**Figure 4:**
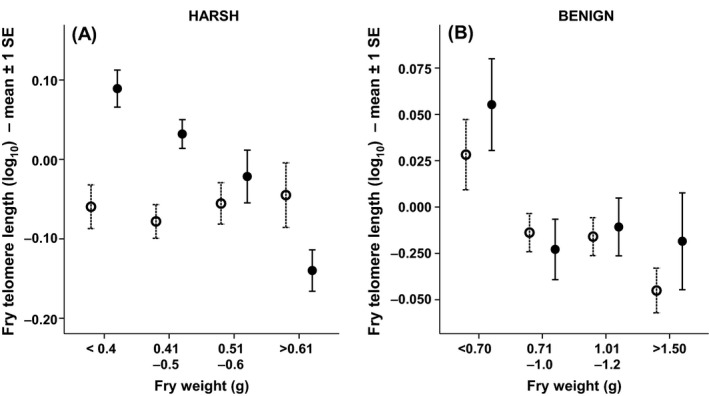
The relationship between fry weight (g) and fry telomere length in (A) the harsh stream and (B) the benign stream. For presentation purposes, open circles represent fry that were captured in areas below the mean fry density (fry/m^2^) and closed circles represent fry that were captured in areas above the mean fry density, although analysis was based on original continuous data. Mean fry density was calculated separately for each stream.

**Figure 5:**
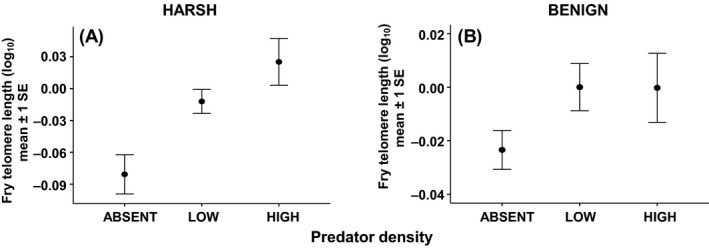
The relationship between predator density and mean fry telomere length in (A) the harsh stream and (B) the benign stream. Predator density categorized for clarity of presentation, but treated as a continuous variable in the analysis. Definitions of predator density: ABSENT = 0, LOW = below the mean salmonid parr density (parr/m^2^), HIGH = above the mean parr density (calculated separately for each stream).

**Figure 6:**
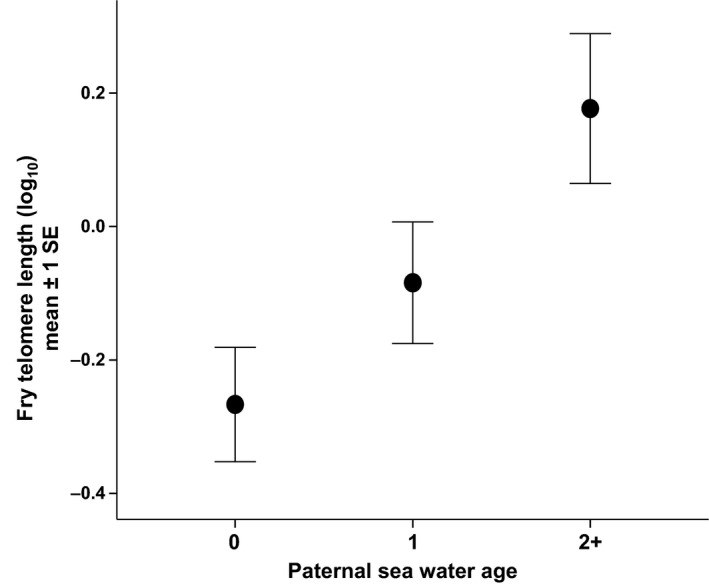
The relationship between paternal sea water age and average fry telomere length. Paternal sea water age definitions: 0 = precocious parr fathers (which have not migrated to sea), 1 = 1SW fathers (which have spent one winter at sea), 2+ = MSW fathers (which have spent 2+ years at sea).

Telomere length in Atlantic salmon fry was negatively correlated with body size (i.e. growth rate), but this effect was dependent on the stream from which they came (significant fry body weight × stream interaction; Table 1D). Faster‐growing fry thus paid a disproportionate cost, in terms of reduced telomere length, in the harsher stream (Fig. 3). In addition to this, growth was also more costly in terms of reduced telomere length when fry were at higher densities (significant fry body weight × fry density interaction; Table 1D, Fig. 4). In addition, fry with the longest telomeres were found in areas with the highest predator density (Table 1D, Fig. 5). Paternal SW age also had a significant effect on fry telomere length, with males that had spent longer at sea prior to reproduction producing fry with relatively longer telomeres (Table 1D, Fig. 6). However, fry telomere length was not related to either paternal FW age or maternal FW or SW ages.

## Discussion

Our study demonstrates the complexity of factors that can influence telomere dynamics in early life. We found that faster growth resulted in shorter telomeres but this effect depended on the relative harshness of the environment. We also found significant paternal effects, with males with longer telomeres producing embryos with shorter telomeres, and males that had spent longer at sea producing fry with relatively longer telomeres.

At the time of field site selection, we based our identification of the harsh and benign river systems on differences in altitude, with the rationale that a higher altitude stream would provide a harsher environment as a consequence of having a lower primary productivity (so lower invertebrate food supply for the fish) and being colder (so further from the optimal temperature for growth). The higher altitude Upper Meig tributary was colder and also had a greater density of predators, than the Allt Goibhre tributary. Together, these factors contribute to making the Upper Meig a harsher environment for growth for young salmon than the Allt Goibhre. As predicted, fry growth rate was faster in the more benign stream. It is possible that the difference in growth rate between the streams was also partially due to the difference in predator density, as an increased predator density may result in reduced foraging (Ward *et al*. 2011). Fry density had a negative effect on fry growth rate in both streams, as would be expected because density‐dependent growth is well established in fish (for salmonid example see Jenkins *et al*. 1999).

Interestingly, embryo telomere length increased during the 18‐day aquarium temperature manipulation, between the age of ~65 and ~83 days postfertilization (Fig. 5). Telomerase is often upregulated during various stages of embryogenesis (e.g. Mantell & Greider 1994; Schaetzlein *et al*. 2004), most likely due to the high cell proliferation rate at this stage (for review see Gomes *et al*. 2010). This may also help explain why we did not see a difference in telomere length between the two temperature treatments, as a higher telomerase expression may help buffer any possible environmental effects. It may also be the case that the 18‐day manipulation was too short a treatment period to produce any effect, especially if there is little embryonic variance in growth rate.

Telomere length in Atlantic salmon fry was negatively correlated with mass gain, but the effect was dependent on the stream they inhabited, with a shorter telomere length for a given mass gain in the harsher stream. This suggests that faster‐growing fry pay a higher cost in terms of telomere length in harsher environments. There are several mechanisms that might individually or collectively underlie this effect. First, environmental temperature may affect trade‐offs between cell division (hyperplasia) and cell growth (hypertrophy) in fishes (reviewed by Arendt 2007). Only hyperplasia has direct consequences for telomeres, as telomere loss occurs at each cell division. In Atlantic salmon, several studies have found warm‐incubated hatchlings to have fewer but larger muscle fibres, indicating that hyperplasia is having a relatively smaller contribution towards growth when salmon develop in warmer conditions (Stickland *et al*. 1988; Usher *et al*. 1994). A possible cause of this effect is that dissolved oxygen is lower at warmer water temperature, which will limit an individual's capacity for cell proliferation (Matschak *et al*. 1997). Therefore, fish developing in warmer water may achieve somatic body growth most efficiently by increasing the size of their existing cells (hypertrophy).

Temperature may also affect the trade‐off between growth and self‐maintenance: individuals that allocate greater resources to growth will have a reduced allocation of resources to self‐maintenance, for example tissue repair and antioxidant production (Zera & Harshman 2001). Temperature has been shown to affect components of the energy budget in the closely related brown trout (*Salmo trutta*), with the amount of energy allocated to body materials (i.e. body growth and body maintenance) decreasing either side of the temperature at which growth is maximized (Elliott 1976). The optimal temperature for growth in juvenile Atlantic salmon is thought to be around 16 °C (Elliott & Hurley 1997) which was only just reached by the warmer (benign) stream by midsummer (the end of the experiment). Therefore, while neither stream provided ideal conditions for growth, the thermal conditions in the harsh stream were further from the optimum. For a given temperature (and food intake), body growth rate can only vary through an alteration of the relative amount of energy allocated to body growth vs body maintenance (McCarthy *et al*. 1994). Therefore, achieving growth at a relatively colder temperature may come at a cost to body maintenance. A reduction in body maintenance (e.g. protein and DNA repair) could in turn affect telomere dynamics.

Ambient temperature may play an additional role in the telomere dynamics of ectotherms as it can affect the exposure of telomeres to oxidative damage through its effect on mitochondrial functioning. Telomeric DNA is vulnerable to oxidative damage from reactive oxygen species (ROS) and most ROS are produced in the mitochondria, which are affected by temperature in several ways. For example, it is often the case in endotherms that mitochondrial density increases as a phenotypic response to lowered temperature (for reviews, see Guderley 2004; O'Brien 2011). In ectotherms, mitochondrial respiration rate (and hence ATP production) decreases at lower temperatures (Schulte 2015), and it may be that an individual compensates by increasing mitochondrial density, thus helping to maintain ATP production (O'Brien 2011). An increase in mitochondrial density may also result in increased ROS production (Crockett 2008). Therefore, fish growing in colder environments might be exposed to more ROS and hence have more telomere attrition.

In addition to identifying a growth environment effect on telomere length between the two streams, we also found growth environment effects within streams. Electrofishing was conducted within 10‐m‐long sections, and for each section, we were able to calculate fry density. Although overall fry density did not differ significantly between the two streams, there was sufficient variation in density between the different sections within each stream to reveal a significant growth fry density effect on telomere length. In the benign stream, fry density had no significant effect on the relationship between fry weight and fry telomere length. However, in the harsh stream, it was beneficial in terms of telomere length for small, but not large fry to be at higher densities. The contrast between streams may reflect resource availability, with the harsher high‐altitude stream likely being more oligotrophic with reduced food availability. There were also more predators in the harsher stream. Therefore, while high densities may confer benefits to smaller fish from increased predator protection, one would expect increased intraspecific competition for resources (e.g. food) as individuals grow larger. Previous studies in other taxa have linked density to physiological stress (Montero *et al*. 1999; Trenzado *et al*. 2006) and telomere attrition (Kotrschal *et al*. 2007; Sohn *et al*. 2012). However, these studies have mostly involved experimental manipulations involving extreme differences in density (crowded vs noncrowded), whereas here the effect was found among naturally occurring variation in density over relatively small spatial scales. Overall, the results are consistent with the hypothesis that growth in more challenging environments comes at a greater cost to telomere length.

There was also a significant predator density effect: areas of higher predator density within each stream were associated with increased fry telomere length. This is somewhat surprising as predation risk has previously been linked to a number of physiological stress parameters (Hawlena & Schmitz 2010; Clinchy *et al*. 2013) including oxidative stress (Slos & Stoks 2008; Janssens & Stoks 2013) and telomere loss (Olsson *et al*. 2010). However, predatory salmonid parr will exhibit greater movement within a stream compared to the limited movement of salmon fry. Therefore, our assessment of predatory parr density may only be a snapshot of parr distribution at the time of electrofishing. With this in mind, a more controlled laboratory manipulation is needed to elucidate potential predator effects on physiological stress pathways and telomere dynamics. The predator densities recorded here may actually have been an index of environmental quality. Predator distribution is partially dependent on prey abundance, and fry abundance would be expected to be greater in good quality habitat (e.g. plentiful shelter and food availability). Therefore, better quality habitats within each stream may be simultaneously producing healthier offspring, with an associated reduction in telomere attrition, while at the same time attracting more predators due to the numbers of fry. Thus, a greater local predator density could actually provide a more benign environment for surviving fry by reducing fry densities and hence intracohort competition, although the situation is complicated by the evidence that fry and older fish may at times compete for the same resources (Kaspersson *et al*. 2012). Notwithstanding this possibility, we did not detect a significant correlation between predator density and fry density.

Offspring telomere length was not affected by any of the maternal traits included in the models. In contrast, however, it was significantly influenced by paternal telomere length and paternal life history. There was a negative relationship between paternal telomere length and offspring (embryo) telomere length. This effect was evident both before and after the temperature manipulation, but the reasons for it are unclear. As there were no maternal effects, and we used an in vitro fertilization design, assortative mating is not involved in generating this pattern, and it might possibly relate to some differences in telomerase activity during the embryo stage, but this would require further investigation. There was a positive relationship between the number of years fathers (but not mothers) had spent at sea (ranging between 0 and 2) and offspring (fry) telomere length. A number of studies report significant paternal inheritance of telomere length, mostly in humans (i.e. De Meyer *et al*. 2007; Njajou *et al*. 2007) but also in lizards (Olsson *et al*. 2011). This effect is often attributed to paternal age, as telomere length has been shown to increase with age in human sperm cells (Allsopp *et al*. 1992). This may help explain our paternal effect in Atlantic salmon because, on average, MSW males will be oldest and precocious parr will be youngest. However, no effect was found of time spent by fathers in fresh water, and these paternal life‐history variants differ in many other ways than just age. Perhaps the most striking difference is in body size: MSW males used in this experiment were >2.5 times heavier than 1SW males and almost 200 times heavier than the precocious parr. If the larger fathers produce offspring that grow faster, their offspring might have been expected to have shorter telomere lengths; this pattern of reduced telomere length in larger individuals has been reported, for example in birds (Ringsby *et al*. 2015). However, our results suggest the opposite pattern, but we cannot separate time spent at sea from age and body size in this study. This variation in size and associated growth rate may be associated with differing levels of telomerase expression. A number of fish studies have detected telomerase activity in various adult tissues (Klapper *et al*. 1998; Hatakeyama *et al*. 2008; Gomes *et al*. 2010). It has also been reported that telomerase expression in fish is positively correlated with cell proliferation (Yap *et al*. 2005; Peterson *et al*. 2015). In fish, muscle fibre recruitment continues beyond embryogenesis (Weatherley *et al*. 1988) and active cell proliferation is still detectable in Atlantic salmon at least 6 months after transfer to sea water (Johnston *et al*. 2003). Therefore, it is possible that the higher growth rate of MSW fish results in a greater expression of telomerase, which in turn may affect germline telomere dynamics, and subsequently offspring telomere dynamics.

This study demonstrates the complexity of parental and environmental factors that can influence telomere dynamics in early life, but highlights the fact that paternal effects may be stronger than maternal and that growth in harsher conditions has greater costs in terms of telomere length. It may be that individuals growing in harsher early environments are required to invest more in telomere maintenance at a later stage, if they are to avoid an acceleration of senescence. However, we now need to examine which features of a harsher environment have the greatest impact on telomere dynamics. Doing so will allow more accurate predictions of the effect of environmental conditions on patterns of senescence.

D.M., J.D.A., S.M., P.M. and N.B.M. contributed to experimental design. D.M., D.C.S. and S.M. carried out the field experiment and D.M. performed the laboratory analysis. Telomere data were analysed by D.M. and W.B. The article was written by D.M., with additional comments from W.B., P.M. and N.B.M.

## Data accessibility

Data deposited in the Dryad repository: doi:10.5061/dryad.2r6r4.

## Supporting information


**Fig. S1** Map outlining the location of (A) the Loch na Croic fish trap; (B) the Allt Goibhre (benign) tributary; (C) the Upper Meig (harsh) tributary and (D) the entrance to the Cromarty firth, which is the mouth of the River Conon catchment in which the two tributaries and trap are located.
**Fig. S2** A schematic diagram of the split‐brood in vitro fertilisation design, utilising all possible parent types with respect to time spent in sea water.
**Fig. S3** Summary of the average temperatures in the two tributary streams over the course of the experiment.
**Table S1** Summary of the SFCC general electrofishing habitat survey results for the two experimental streams.
**Table S2** Summary of the initial full linear mixed‐effect model explaining variation in fry weight (g).
**Table S3** Summary of the initial full linear mixed‐effect model explaining variation in fry survival.
**Table S4** Summary of the initial full linear mixed‐effect model explaining variation in embryo telomere length.
**Table S5** Summary of the initial full linear mixed‐effect model explaining variation in fry telomere length.
**Fig. S4** Comparison of the mean fry weight (g) in the two streams at the time of recapture, approximately 2 months after first feeding.
**Fig. S5** The relationship between fry density and fry weight (g).
**Fig. S6** The relationship between average dry egg weight per family (g) and subsequent fry weight (g) at the time of recapture.
**Fig. S7** The relationship between average dry egg weight per family (g) and subsequent fry survival.
**Fig. S8** The relationship between paternal telomere length and embryo telomere length.

## References

[mec13857-bib-0001] Allsopp RC , Vaziri H , Patterson C *et al* (1992) Telomere length predicts replicative capacity of human fibroblasts. Proceedings of the National Academy of Sciences, USA, 89, 10114–10118. 10.1073/pnas.89.21.10114 PMC50288 1438199

[mec13857-bib-0002] Alonso‐Álvarez C , Bertrand S , Faivre B , Sorci G (2007) Increased susceptibility to oxidative damage as a cost of accelerated somatic growth in zebra finches. Functional Ecology, 21, 873–879.

[mec13857-bib-0003] Arendt J (2007) Ecological correlates of body size in relation to cell size and cell number: patterns in flies, fish, fruits and foliage. Biological Reviews, 82, 241–256. 17437559 10.1111/j.1469-185X.2007.00013.x

[mec13857-bib-0004] Asghar M , Bensch S , Tarka M , Hansson B , Hasselquist D (2015) Maternal and genetic factors determine early life telomere length. Proceedings of the Royal Society of London B: Biological Sciences, 282, 20142263. 10.1098/rspb.2014.2263 PMC4286038 25621325

[mec13857-bib-0005] Baum D , Laughton R , Armstrong JD , Metcalfe NB (2004) Altitudinal variation in the relationship between growth and maturation rate in salmon parr. Journal of Animal Ecology, 73, 253–260.

[mec13857-bib-0006] Blackburn EH (1991) Structure and function of telomeres. Nature, 350, 569–573. 1708110 10.1038/350569a0

[mec13857-bib-0007] Blanckenhorn WU (2000) The evolution of body size: what keeps organisms small? The Quarterly Review of Biology, 75, 385–407. 11125698 10.1086/393620

[mec13857-bib-0008] Burton T , McKelvey S , Stewart D , Armstrong J , Metcalfe N (2013) Early maternal experience shapes offspring performance in the wild. Ecology, 94, 618–626. 23687888 10.1890/12-0462.1

[mec13857-bib-0009] Calado RT , Young NS (2009) Telomere diseases. New England Journal of Medicine, 361, 2353–2365. 20007561 10.1056/NEJMra0903373 PMC3401586

[mec13857-bib-0010] Campisi J , S‐H Kim , Lim C‐S , Rubio M (2001) Cellular senescence, cancer and aging: the telomere connection. Experimental Gerontology, 36, 1619–1637. 11672984 10.1016/s0531-5565(01)00160-7

[mec13857-bib-0011] Carney Almroth B , Johnsson JI , Devlin R , Sturve J (2012) Oxidative stress in growth hormone transgenic coho salmon with compressed lifespan–a model for addressing aging. Free radical research, 46, 1183–1189. 22655913 10.3109/10715762.2012.698009

[mec13857-bib-0012] Cawthon RM (2002) Telomere measurement by quantitative PCR. Nucleic Acids Research, 30, e47. 12000852 10.1093/nar/30.10.e47 PMC115301

[mec13857-bib-0013] Chan SRWL , Blackburn EH (2004) Telomeres and telomerase. Philosophical Transactions of the Royal Society B: Biological Sciences, 359, 109–121. 10.1098/rstb.2003.1370 PMC1693310 15065663

[mec13857-bib-0014] Clinchy M , Sheriff MJ , Zanette LY (2013) Predator‐induced stress and the ecology of fear. Functional Ecology, 27, 56–65.

[mec13857-bib-0015] Collins K (2006) The biogenesis and regulation of telomerase holoenzymes. Nature Reviews Molecular Cell Biology, 7, 484–494. 16829980 10.1038/nrm1961 PMC2915765

[mec13857-bib-0016] Crockett EL (2008) The cold but not hard fats in ectotherms: consequences of lipid restructuring on susceptibility of biological membranes to peroxidation, a review. Journal of Comparative Physiology B, 178, 795–809. 10.1007/s00360-008-0275-7 18506451

[mec13857-bib-0017] De Meyer T , Rietzschel ER , De Buyzere ML *et al* (2007) Paternal age at birth is an important determinant of offspring telomere length. Human Molecular Genetics, 16, 3097–3102. 17881651 10.1093/hmg/ddm271

[mec13857-bib-0018] Dmitriew CM (2011) The evolution of growth trajectories: what limits growth rate? Biological Reviews, 86, 97–116. 20394607 10.1111/j.1469-185X.2010.00136.x

[mec13857-bib-0019] Eisenberg DTA (2014) Inconsistent inheritance of telomere length (TL): is offspring TL more strongly correlated with maternal or paternal TL. European Journal of Human Genetics, 22, 8–9. 24022299 10.1038/ejhg.2013.202 PMC3865394

[mec13857-bib-0020] Elliott JM (1976) Body composition of brown trout (*Salmo trutta* L.) in relation to temperature and ration size. Journal of Animal Ecology, 45, 273–289.

[mec13857-bib-0021] Elliott JM , Hurley MA (1997) A functional model for maximum growth of Atlantic salmon parr, *Salmo salar*, from two populations in northwest England. Functional Ecology, 11, 592–603.

[mec13857-bib-0022] Elliott SR , Coe TA , Helfield JM , Naiman RJ (1998) Spatial variation in environmental characteristics of Atlantic salmon (Salmo salar) rivers. Canadian Journal of Fisheries and Aquatic Sciences, 55, 267–280.

[mec13857-bib-0023] Fick LJ , Fick GH , Li Z *et al* (2012) Telomere length correlates with life span of dog breeds. Cell Reports, 2, 1530–1536. 23260664 10.1016/j.celrep.2012.11.021

[mec13857-bib-0024] Fleming IA (1996) Reproductive strategies of Atlantic salmon: ecology and evolution. Reviews in Fish Biology and Fisheries, 6, 379–416.

[mec13857-bib-0025] Geiger S , Le Vaillant M , Lebard T *et al* (2012) Catching‐up but telomere loss: half‐opening the black box of growth and ageing trade‐off in wild king penguin chicks. Molecular Ecology, 21, 1500–1510. 22117889 10.1111/j.1365-294X.2011.05331.x

[mec13857-bib-0026] Gomes NMV , Shay JW , Wright WE (2010) Telomere biology in Metazoa. FEBS Letters, 584, 3741–3751. 20655915 10.1016/j.febslet.2010.07.031 PMC2928394

[mec13857-bib-0027] Guderley H (2004) Metabolic responses to low temperature in fish muscle. Biological Reviews, 79, 409–427. 15191230 10.1017/s1464793103006328

[mec13857-bib-0028] Halliwell B , Gutteridge JM (2015) Free Radicals in Biology and Medicine. Oxford University Press, Oxford.

[mec13857-bib-0029] Hatakeyama H , Nakamura KI , Izumiyama‐Shimomura N *et al* (2008) The teleost *Oryzias latipes* shows telomere shortening with age despite considerable telomerase activity throughout life. Mechanisms of Ageing and Development, 129, 550–557. 18597819 10.1016/j.mad.2008.05.006

[mec13857-bib-0030] Haussmann MF , Marchetto NM (2010) Telomeres: linking stress and survival, ecology and evolution. Current Zoology, 56, 714–727.

[mec13857-bib-0031] Haussmann MF , Winkler DW , Vleck CM (2005) Longer telomeres associated with higher survival in birds. Biology Letters, 1, 212–214. 17148169 10.1098/rsbl.2005.0301 PMC1626238

[mec13857-bib-0032] Haussmann MF , Longenecker AS , Marchetto NM , Juliano SA , Bowden RM (2011) Embryonic exposure to corticosterone modifies the juvenile stress response, oxidative stress and telomere length. Proceedings of the Royal Society of London B: Biological Sciences, 279, 1447–1456. 10.1098/rspb.2011.1913 PMC3282378 22072607

[mec13857-bib-0033] Hawlena D , Schmitz OJ (2010) Physiological stress as a fundamental mechanism linking predation to ecosystem functioning. The American Naturalist, 176, 537–556. 10.1086/656495 20846014

[mec13857-bib-0034] Heidinger BJ , Blount JD , Boner W *et al* (2012) Telomere length in early life predicts lifespan. Proceedings of the National Academy of Sciences, USA, 109, 1743–1748. 10.1073/pnas.1113306109 PMC3277142 22232671

[mec13857-bib-0035] Hellemans J , Mortier G , De Paepe A , Speleman F , Vandesompele J (2007) qBase relative quantification framework and software for management and automated analysis of real‐time quantitative PCR data. Genome biology, 8, R19. 17291332 10.1186/gb-2007-8-2-r19 PMC1852402

[mec13857-bib-0036] Henderson JN , Letcher BH (2003) Predation on stocked Atlantic salmon (*Salmo salar*) fry. Canadian Journal of Fisheries and Aquatic Sciences, 60, 32–42.

[mec13857-bib-0037] Herborn KA , Heidinger BJ , Boner W *et al* (2014) Stress exposure in early post‐natal life reduces telomere length: an experimental demonstration in a long‐lived seabird. Proceedings of the Royal Society of London B: Biological Sciences, 281, 20133151. 10.1098/rspb.2013.3151 PMC3973262 24648221

[mec13857-bib-0038] Horn T , Robertson BC , Will M *et al* (2011) Inheritance of telomere length in a bird. PLoS One, 6, e17199. 21364951 10.1371/journal.pone.0017199 PMC3043093

[mec13857-bib-0039] Ilmonen P , Kotrschal A , Penn DJ (2008) Telomere attrition due to infection. PLoS One, 3, e2143. 18478110 10.1371/journal.pone.0002143 PMC2366059

[mec13857-bib-0040] Janssens L , Stoks R (2013) Predation risk causes oxidative damage in prey. Biology Letters, 9, 0130350. 10.1098/rsbl.2013.0350 PMC3730648 23760170

[mec13857-bib-0041] Jenkins TMJ , Diehl S , Kratz KW , Cooper SD (1999) Effects of population density on individual growth of brown trout in streams. Ecology, 80, 941–956.

[mec13857-bib-0042] Johnston IA (2006) Environment and plasticity of myogenesis in teleost fish. Journal of Experimental Biology, 209, 2249–2264. 16731802 10.1242/jeb.02153

[mec13857-bib-0043] Johnston IA , Manthri S , Alderson R *et al* (2003) Freshwater environment affects growth rate and muscle fibre recruitment in seawater stages of Atlantic salmon (*Salmo salar* L.). Journal of Experimental Biology, 206, 1337–1351. 12624169 10.1242/jeb.00262

[mec13857-bib-0044] Jonsson B , Jonsson N (2011) Ecology of Atlantic Salmon and Brown Trout: Habitat as a Template for Life Histories, 1st edn Springer, Dordrecht, the Netherlands.

[mec13857-bib-0045] Jonsson B , Forseth T , Jensen AJ , Næsje TF (2001) Thermal performance of juvenile Atlantic salmon, *Salmo salar* L. Functional Ecology, 15, 701–711.

[mec13857-bib-0046] Kaspersson R , Höjesjö J , Bohlin T (2012) Habitat exclusion and reduced growth: a field experiment on the effects of inter‐cohort competition in young‐of‐the‐year brown trout. Oecologia, 169, 733–742. 22271199 10.1007/s00442-012-2248-5

[mec13857-bib-0047] Kim S‐Y , Velando A (2015) Antioxidants safeguard telomeres in bold chicks. Biology Letters, 11, 20150211. 25948570 10.1098/rsbl.2015.0211 PMC4455747

[mec13857-bib-0048] Klapper W , Heidorn K , Kuhne K , Parwaresch R , Krupp G (1998) Telomerase activity in ‘immortal’ fish. FEBS Letters, 434, 409–412. 9742964 10.1016/s0014-5793(98)01020-5

[mec13857-bib-0049] Klemetsen A , Amundsen PA , Dempson JB *et al* (2003) Atlantic salmon *Salmo salar* L., brown trout *Salmo trutta* L. and Arctic charr *Salvelinus alpinus* (L.): a review of aspects of their life histories. Ecology of Freshwater Fish, 12, 1–59.

[mec13857-bib-0050] Kotrschal A , Ilmonen P , Penn DJ (2007) Stress impacts telomere dynamics. Biology Letters, 3, 128–130. 17264051 10.1098/rsbl.2006.0594 PMC2375929

[mec13857-bib-0051] Mantell LL , Greider CW (1994) Telomerase activity in germline and embryonic cells of Xenopus. The EMBO Journal, 13, 3211–3217. 8039513 10.1002/j.1460-2075.1994.tb06620.x PMC395213

[mec13857-bib-0052] Marchetto NM , Glynn RA , Ferry ML *et al* (2016) Prenatal stress and newborn telomere length. American Journal of Obstetrics and Gynecology, 215, 94–95. 26829506 10.1016/j.ajog.2016.01.177

[mec13857-bib-0053] Matschak TW , Stickland NC , Mason PS , Crook AR (1997) Oxygen availability and temperature affect embryonic muscle development in Atlantic salmon (*Salmo salar* L.). Differentiation, 61, 229–235.

[mec13857-bib-0054] McCarthy ID , Houlihan DF , Carter CG (1994) Individual variation in protein turnover and growth efficiency in rainbow trout, *Oncorhynchus mykiss* (Walbaum). Proceedings of the Royal Society of London B: Biological Sciences, 257, 141–147.

[mec13857-bib-0055] Metcalfe NB , Monaghan P (2001) Compensation for a bad start: grow now, pay later? Trends in Ecology & Evolution, 16, 254–260. 11301155 10.1016/s0169-5347(01)02124-3

[mec13857-bib-0056] Metcalfe NB , Monaghan P (2003) Growth versus lifespan: perspectives from evolutionary ecology. Experimental Gerontology, 38, 935–940. 12954479 10.1016/s0531-5565(03)00159-1

[mec13857-bib-0057] Metcalfe NB , Thorpe JE (1990) Determinants of geographical variation in the age of seaward‐migrating salmon, *Salmo salar* . Journal of Animal Ecology, 59, 135–145.

[mec13857-bib-0058] Montero D , Izquierdo M , Tort L , Robaina L , Vergara J (1999) High stocking density produces crowding stress altering some physiological and biochemical parameters in gilthead seabream, *Sparus aurata*, juveniles. Fish Physiology and Biochemistry, 20, 53–60.

[mec13857-bib-0059] Naslund J , Pauliny A , Blomqvist D , Johnsson JI (2015) Telomere dynamics in wild brown trout: effects of compensatory growth and early growth investment. Oecologia, 177, 1221–1230. 25698140 10.1007/s00442-015-3263-0

[mec13857-bib-0060] Nettle D , Monaghan P , Gillespie R *et al* (2015) An experimental demonstration that early‐life competitive disadvantage accelerates telomere loss. Proceedings of the Royal Society of London B: Biological Sciences, 282, 20141610. 10.1098/rspb.2014.1610 PMC4262165 25411450

[mec13857-bib-0061] Nislow K , Armstrong J , McKelvey S (2004) Phosphorus flux due to Atlantic salmon (*Salmo salar*) in an oligotrophic upland stream: effects of management and demography. Canadian Journal of Fisheries and Aquatic Sciences, 61, 2401–2410.

[mec13857-bib-0062] Njajou OT , Cawthon RM , Damcott CM *et al* (2007) Telomere length is paternally inherited and is associated with parental lifespan. Proceedings of the National Academy of Sciences, USA, 104, 12135–12139. 10.1073/pnas.0702703104 PMC1924539 17623782

[mec13857-bib-0063] Noguera JC , Metcalfe NB , Boner W , Monaghan P (2015) Sex‐dependent effects of nutrition on telomere dynamics in zebra finches (*Taeniopygia guttata*). Biology Letters, 11, 20140938. 25716087 10.1098/rsbl.2014.0938 PMC4360105

[mec13857-bib-0064] Nordfjall K , Larefalk A , Lindgren P , Holmberg D , Roos G (2005) Telomere length and heredity: indications of paternal inheritance. Proceedings of the National Academy of Sciences, USA, 102, 16374–16378. 10.1073/pnas.0501724102 PMC1283414 16258070

[mec13857-bib-0065] Nussey DH , Pemberton JM , Pilkington JG , Blount JD (2009) Life history correlates of oxidative damage in a free‐living mammal population. Functional Ecology, 23, 809–817.

[mec13857-bib-0066] O'Brien KM (2011) Mitochondrial biogenesis in cold‐bodied fishes. Journal of Experimental Biology, 214, 275–285. 21177947 10.1242/jeb.046854

[mec13857-bib-0067] Økland F , Jonsson B , Jensen AJ , Hansen LP (1993) Is there a threshold size regulating seaward migration of brown trout and Atlantic salmon. Journal of Fish Biology, 42, 541–550.

[mec13857-bib-0068] Olsson M , Pauliny A , Wapstra E , Blomqvist D (2010) Proximate determinants of telomere length in sand lizards (*Lacerta agilis*). Biology Letters, 6, 651–653. 20356883 10.1098/rsbl.2010.0126 PMC2936144

[mec13857-bib-0069] Olsson M , Pauliny A , Wapstra E *et al* (2011) Sex differences in sand lizard telomere inheritance: paternal epigenetic effects increases telomere heritability and offspring survival. PLoS One, 6, e17473. 21526170 10.1371/journal.pone.0017473 PMC3081292

[mec13857-bib-0070] Pauliny A , Devlin RH , Johnsson JI , Blomqvist D (2015) Rapid growth accelerates telomere attrition in a transgenic fish. BMC Evolutionary Biology, 15, 159. 26268318 10.1186/s12862-015-0436-8 PMC4535669

[mec13857-bib-0071] Peterson DR , Mok HO , Au DW (2015) Modulation of telomerase activity in fish muscle by biological and environmental factors. Comparative Biochemistry and Physiology, Part C: Toxicology & Pharmacology, 178, 51–59. 26400776 10.1016/j.cbpc.2015.09.004

[mec13857-bib-0072] Reichert S , Rojas ER , Zahn S *et al* (2015) Maternal telomere length inheritance in the king penguin. Heredity, 114, 10–16. 25052413 10.1038/hdy.2014.60 PMC4815600

[mec13857-bib-0073] Ringsby TH , Jensen H , Pärn H *et al* (2015) On being the right size: increased body size is associated with reduced telomere length under natural conditions. Proceedings of the Royal Society B: Biological Sciences, 282, 20152331. 26631569 10.1098/rspb.2015.2331 PMC4685786

[mec13857-bib-0074] Schaetzlein S , Lucas‐Hahn A , Lemme E *et al* (2004) Telomere length is reset during early mammalian embryogenesis. Proceedings of the National Academy of Sciences, USA, 101, 8034–8038. 10.1073/pnas.0402400101 PMC419552 15148368

[mec13857-bib-0075] Schulte PM (2015) The effects of temperature on aerobic metabolism: towards a mechanistic understanding of the responses of ectotherms to a changing environment. Journal of Experimental Biology, 218, 1856–1866. 26085663 10.1242/jeb.118851

[mec13857-bib-0076] Shearer WM (1992) Atlantic Salmon Scale Reading Guidelines. International Council for the Exploration of the Sea Copenhagen, Copenhagen.

[mec13857-bib-0077] Slos S , Stoks R (2008) Predation risk induces stress proteins and reduces antioxidant defense. Functional Ecology, 22, 637–642.

[mec13857-bib-0078] Sohn SH , Subramani VK , Moon YS , Jang IS (2012) Telomeric DNA quantity, DNA damage, and heat shock protein gene expression as physiological stress markers in chickens. Poultry Science, 91, 829–836. 10.3382/ps.2011-01904 22399721

[mec13857-bib-0079] Stickland NC , White RN , Mescall PE , Crook AR , Thorpe JE (1988) The effect of temperature on myogenesis in embryonic‐development of the Atlantic salmon (*Salmo salar*). Anatomy and Embryology, 178, 253–257. 3414977 10.1007/BF00318228

[mec13857-bib-0080] Stier A , Delestrade A , Zahn S *et al* (2014) Elevation impacts the balance between growth and oxidative stress in coal tits. Oecologia, 175, 791–800. 24805201 10.1007/s00442-014-2946-2

[mec13857-bib-0081] Stindl R (2004) Tying it all together: telomeres, sexual size dimorphism and the gender gap in life expectancy. Medical Hypotheses, 62, 151–154. 14729022 10.1016/s0306-9877(03)00316-5

[mec13857-bib-0082] Trenzado CE , Morales AE , de la Higuera M (2006) Physiological effects of crowding in rainbow trout, *Oncorhynchus mykiss*, selected for low and high stress responsiveness. Aquaculture, 258, 583–593.

[mec13857-bib-0083] Usher ML , Stickland NC , Thorpe JE (1994) Muscle development in Atlantic salmon (*Salmo salar*) embryos and the effect of temperature on muscle cellularity. Journal of Fish Biology, 44, 953–964.

[mec13857-bib-0084] Ward DM , Nislow KH , Folt CL (2011) Seasonal shift in the effects of predators on juvenile Atlantic salmon (*Salmo salar*) energetics. Canadian Journal of Fisheries and Aquatic Sciences, 68, 2080–2089. 10.1139/f2011-123 PMC5089841 27812237

[mec13857-bib-0085] Weatherley AH , Gill HS , Lobo AF (1988) Recruitment and maximal diameter of axial muscle fibres in teleosts and their relationship to somatic growth and ultimate size. Journal of Fish Biology, 33, 851–859.

[mec13857-bib-0086] Yap WH , Yeoh E , Brenner S , Venkatesh B (2005) Cloning and expression of the reverse transcriptase component of pufferfish (*Fugu rubripes*) telomerase. Gene, 353, 207–217. 15961261 10.1016/j.gene.2005.04.038

[mec13857-bib-0087] Zera AJ , Harshman LG (2001) The physiology of life history trade‐offs in animals. Annual Review of Ecology and Systematics, 32, 95–126.

[mec13857-bib-0088] von Zglinicki T (2002) Oxidative stress shortens telomeres. Trends in Biochemical Sciences, 27, 339–344. 12114022 10.1016/s0968-0004(02)02110-2

