# Supplementary material for: Interactions between parental traits, environmental harshness and growth rate in determining telomere length in wild juvenile salmon
Source: Mol Ecol. 2016 Oct 14;25(21):5425–38. doi: 10.1111/mec.13857 (PMC5091633; doi:10.1111/mec.13857)
Supplement: Supplementary file 1 — Fig. S1 Map outlining the location of (A) the Loch na Croic fish trap; (B) the Allt Goibhre (benign) tributary; (C) the Upper Meig (harsh) tributary and (D) the entrance to the Cromarty firth, which is the mouth of the River Conon catchment in which the two tributaries and trap are located. Fig. S2 A schematic diagram of the split‐brood in vitro fertilisation design, utilising all possible parent types with respect to time spent in sea water. Fig. S3 Summary of the average temperatures in the two tributary streams over the course of the experiment. Table S1 Summary of the SFCC general electrofishing habitat survey results for the two experimental streams. Table S2 Summary of the initial full linear mixed‐effect model explaining variation in fry weight (g). Table S3 Summary of the initial full linear mixed‐effect model explaining variation in fry survival. Table S4 Summary of the initial full linear mixed‐effect model explaining variation in embryo telomere length. Table S5 Summary of the initial full linear mixed‐effect model explaining variation in fry telomere length. Fig. S4 Comparison of the mean fry weight (g) in the two streams at the time of recapture, approximately 2 months after first feeding. Fig. S5 The relationship between fry density and fry weight (g). Fig. S6 The relationship between average dry egg weight per family (g) and subsequent fry weight (g) at the time of recapture. Fig. S7 The relationship between average dry egg weight per family (g) and subsequent fry survival. Fig. S8 The relationship between paternal telomere length and embryo telomere length. [file MEC-25-5425-s001.docx]

**SUPPORTING INFORMATION**

**SUPPORTING METHODS**


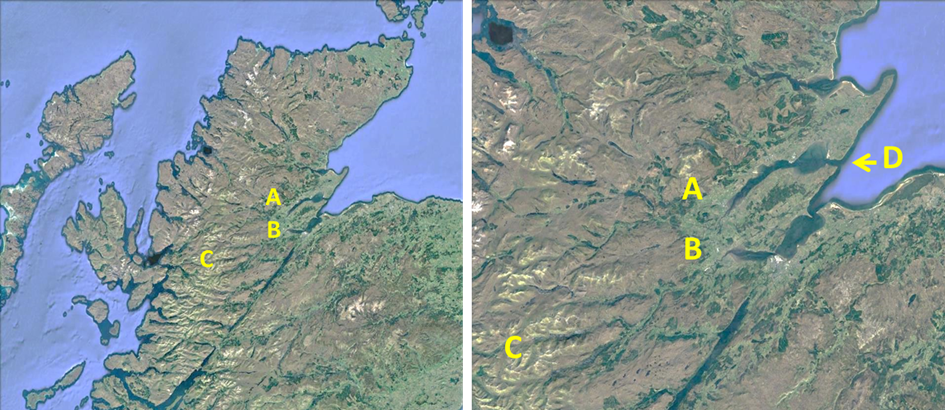


**Figure S1. Map outlining the location of (A) the Loch na Croic fish trap; (B) the Allt Goibhre (benign) tributary; (C) the Upper Meig (harsh) tributary and (D) the entrance to the Cromarty firth, which is the mouth of the River Conon catchment in which the two tributaries and trap are located.**

**
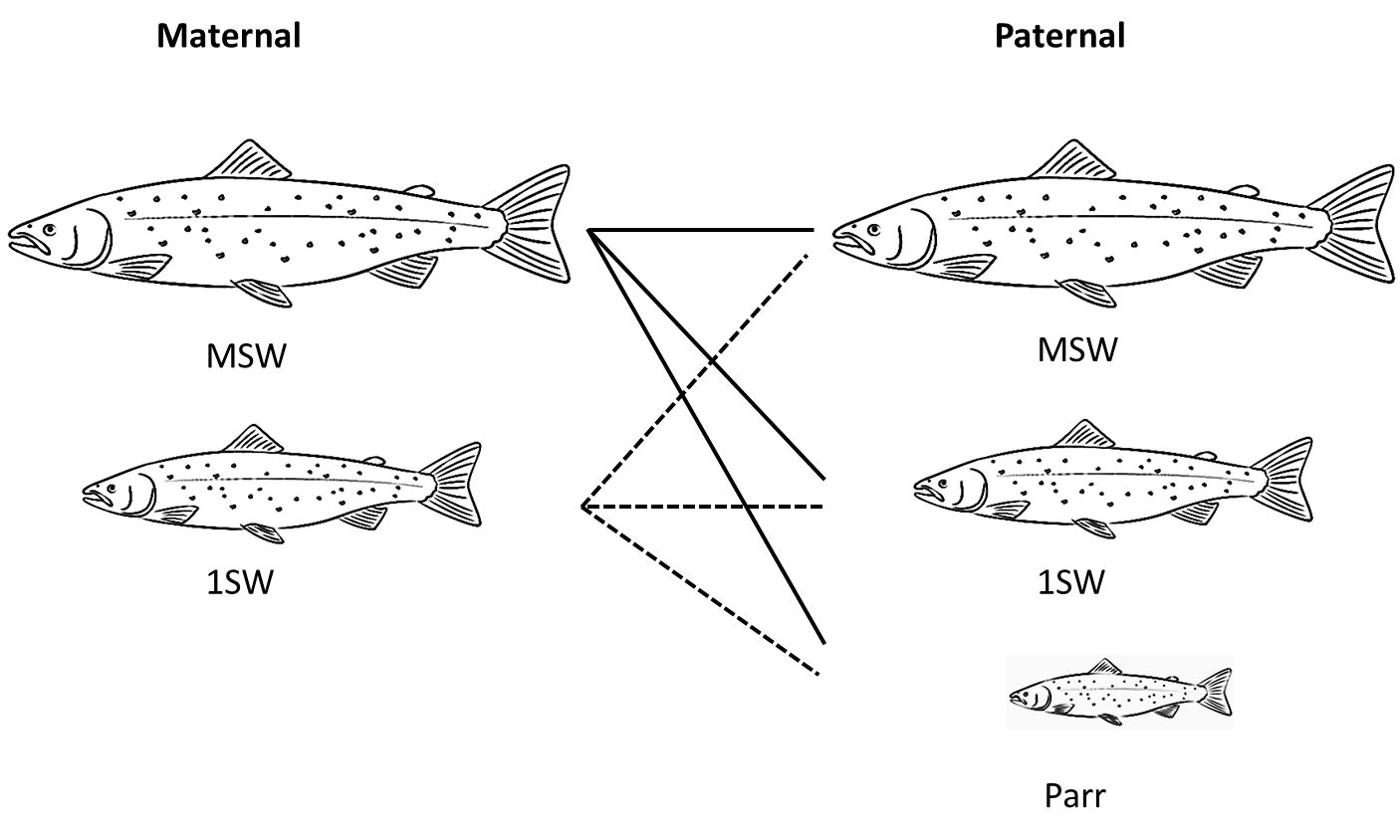
**

**Figure S2. A schematic diagram of the split-brood *in vitro* fertilisation design, utilising all possible parent types with respect to time spent in sea water**. 1SW = 1 year spent in sea water, MSW = 2+ years spent in sea water and Parr = males that matured in FW and spent 0 years in sea water.

**
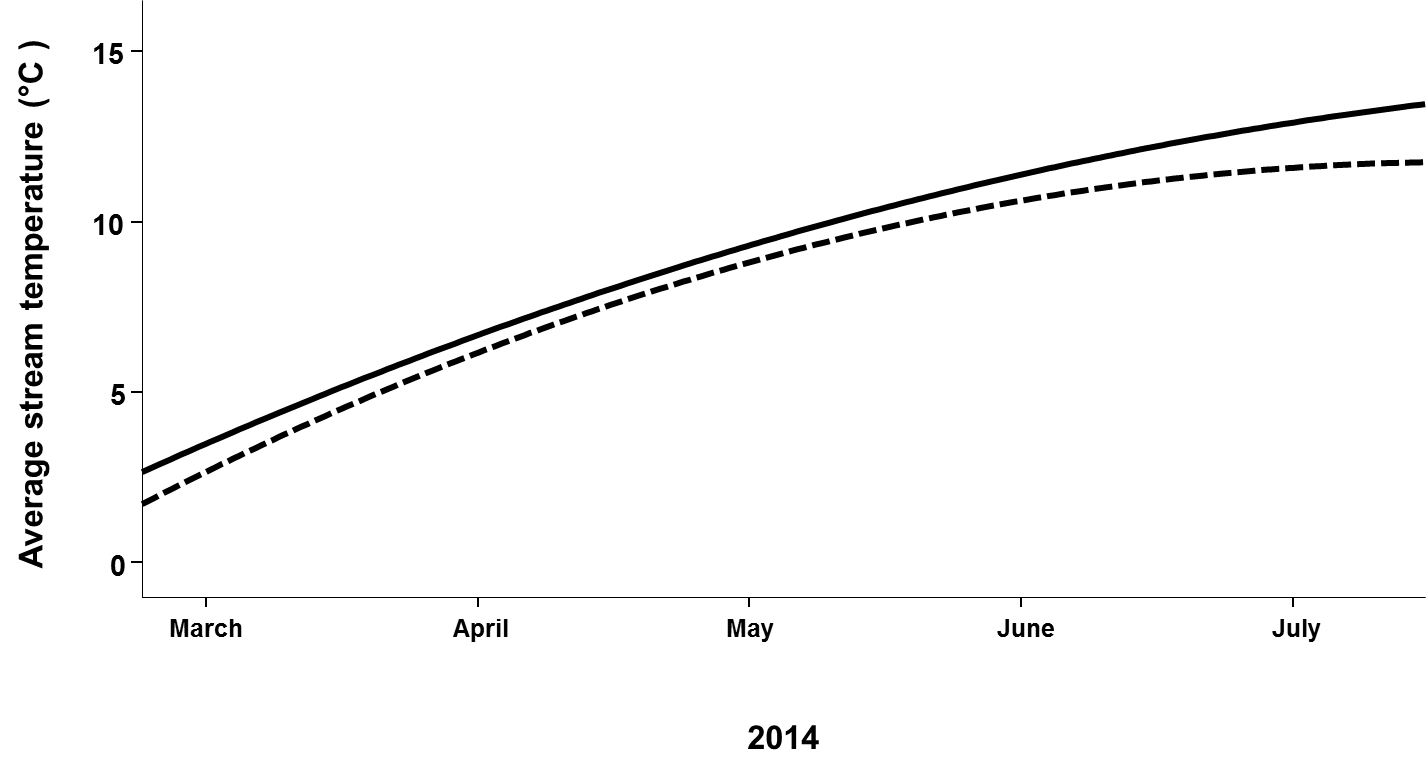
**

**Figure S3. Summary of the average temperatures in the two tributary streams over the course of the experiment**. HOBO temperature data loggers were placed in each stream at the time and site of egg deposition and programmed to record data every 4h. For presentation purposes, data have been fitted to a quadratic regression line and individual data points have been removed. Solid line = Allt Goibhre (benign) stream, in which eggs were planted on February 26^th^ and fry were recaptured on 23^rd^ July. Dashed line = Upper Meig (harsh) stream, in which eggs were planted on March 1^st^ and fry recaptured on July 22^nd^.

|  | **Harsh** | | **Benign** | | **Difference between sites** | | |
| --- | --- | --- | --- | --- | --- | --- | --- |
|  | **Mean** | **s.e.** | **Mean** | **s.e.** | **T** | **d.f.** | **p** |
| **Mean temp. (°C)** | 8.48 | 0.63 | 9.20 | 0.65 | 4.88 | 19 | <0.001 |
| **Fry density (fry/m^2^)** | 0.75 | 0.08 | 0.75 | 0.08 | 0.029 | 29 | 0.977 |
| **Parr density (parr/m^2^)** | 0.11 | 0.02 | 0.03 | 0.01 | 3.476 | 25.08 | 0.002 |
|  |  |  |  |  |  |  |  |
| ***% Depth (cm)*** |  |  |  |  |  |  |  |
| 0-10 | 34.5 | 4.59 | 27.73 | 6.99 | 0.841 | 29 | 0.408 |
| 11-20 | 40 | 4.63 | 48.64 | 4.91 | -1.192 | 29 | 0.243 |
| 21-30 | 22.3 | 3.47 | 19.55 | 5.82 | 0.426 | 29 | 0.673 |
| 31-40 | 3.0 | 1.05 | 4.09 | 2.00 | -0.533 | 29 | 0.598 |
| 41-50 | 0.25 | 0.25 | 0.00 | 0.00 | 0.736 | 29 | 0.468 |
|  |  |  |  |  |  |  |  |
| ***% Substrate*** |  |  |  |  |  |  |  |
| Gravel | 5.5 | 0.80 | 5.9 | 0.91 | -0.320 | 29 | 0.751 |
| Pebble | 40.25 | 4.61 | 22.27 | 2.06 | 3.560 | 25.44 | 0.001 |
| Cobble | 44.0 | 4.22 | 47.27 | 2.64 | -0.540 | 29 | 0.593 |
| Boulder | 10.25 | 2.16 | 24.55 | 2.82 | -3.987 | 29 | <0.001 |
|  |  |  |  |  |  |  |  |
| ***% Flow*** |  |  |  |  |  |  |  |
| Riffle | 41.58 | 6.19 | 45.90 | 8.52 | -0.416 | 28 | 0.680 |
| Run | 12.89 | 4.59 | 0.91 | 0.91 | 2.560 | 19.384 | 0.019 |
| Glide | 44.47 | 5.77 | 50.91 | 8.50 | -0.646 | 28 | 0.523 |
| Pool | 1.05 | 0.61 | 2.27 | 1.04 | -1.084 | 28 | 0.288 |

**Table S1: Summary of the SFCC general electrofishing habitat survey results for the two experimental streams.** Definitions: Gravel = inorganic particles 2-16mm. Pebble = inorganic particles 16-64mm. Cobble = inorganic particle 64-256mm. Boulder = inorganic particle >256mm. Riffle = fast, broken waves, audible. Run = fast, unbroken waves, silent. Glide = moderate/fast, smooth, silent. Pool = slow, eddy, pool. We used a Paired-Sample t test to analyse the difference in temperature at each time point between the two streams. For all other habitat variables, we used Independent-Sample t-tests to analyse the difference between streams.

**Egg planting**

The eggs were planted in pairs of artificial nests of c.500 eggs at 20 different sites along a section of each stream; sites were 10-20m apart, depending on stream width and the availability of suitable spawning substrate and water flow. Artificial nests were made by excavating a hole (at least 20cm deep) in clean well oxygenated substrate. A pipe (10cm diameter) was placed in the centre of the hole and substrate was replaced around the pipe, to form the basic structure of the nest. Eggs were then poured down the pipe, into the centre of the nest. The pipe was then carefully removed and more substrate was added to cover the nest. These artificial nests were similar in structure to natural nests (Aas *et al.* 2011; Jonsson & Jonsson 2011). A Vibert box (containing c.100 eggs) was used at the first, middle and last site, in addition to the two artificial nests, to assess hatching success on each river. The Vibert boxes were recovered in May 2014 and any unhatched eggs were counted. No unhatched eggs were present in any of the Vibert boxes, which indicate an excellent hatching rate for each of the streams.

**Tissue lysis**

For the parental adipose fin tissue, a small sample of tissue was dissected and placed in 180µl Buffer ATL + 20µl proteinase K solution. Samples were incubated at 56°C until the tissue was fully lysed. For the eyed embryo tissue (both pre- and post- aquarium temperature treatment), 5 embryos per family were dissected from the surrounding lipid mass, pooled and homogenised in 1ml of 1 x PBS in a 2ml tube containing a ceramic bead. 100µl of the homogenate was then added to 80µl Buffer ATL + 20µl proteinase K. Samples were incubated at 56°C until the tissue was fully lysed. For the fry tissue, the adipose fin was carefully dissected from each fry and added to 180µl Buffer ATL + 20µl proteinase K. Samples were incubated at 56°C until the tissue was fully lysed. Each set of DNA extractions conducted also included a negative control which contained all of the reagents, but without any tissue. This was used to check for contamination during the lysis and extraction steps. DNA concentration and purity was measured spectrophotometrically using a Nanodrop 8000.

**Primer development**

The Salmo salar GAPDH sequence (Genbank accession number: NM_001123561) was used to design primers for the single copy gene (S) assay. Using the Genbank blast tool, the gene was compared to the Atlantic salmon genome to confirm that it was present at a single location. The following forward and reverse GAPDH primers successfully amplified a single amplicon; as determined by melt curve analysis and were subsequently used in the S assay:

salGAP8-F 5’-GTAAGACAGGATTGAGGCATCTC-3’ and

salGAP8-R 5’-CCGAATCCATTGACACCTACTT-3’

For amplification of telomeric repeats (T assay) the universal primers designed by Cawthon (2002) were used:

Tel1b 5’-CGGTTTGTTTGGGTTTGGGTTTGGGTTTGGGTTTGGGTT-3’ and

Tel2b 5’-GGCTTGCCTTACCCTTACCCTTACCCTTACCCTTACCCT-3’.

A primer optimisation matrix was used to determine the optimal concentration of the primers (for both S and T assay) and five different samples of Atlantic salmon DNA were tested to confirm primer performance and specificity.

**Genotyping**

Genotyping was conducted by commercial suppliers (Landcatch Natural Selection Ltd, Stirling, Scotland) using a panel of markers that they had customised for internal use. DNA was extracted from the fin clips of all parental fish and recaptured offspring using an E-Z 96 tissue DNA Tissue kit (Omega Bio-Tek, Georgia, USA) following the manufacturer’s protocol. Genotyping was performed using three iplex panels, which equalled 106 informative SNPs scattered across the genome. A Sequenom MassARRAY platform (Sequenom Inc., California, USA) was used for the genotyping of the samples. Analysis for parentage assignment by exclusion was carried out with the programme Vitassign 8.3 (Vandeputte *et al.* 2006) with some modifications to allow the analysis of more than 100 markers. From a total of 843 fry, 822 (>97%) were uniquely assigned to a single set of parents, 8 had a Single Parental Assignment (0.95%) and 13 failed to assign, mostly due to low allele calls.

|  | **Numerator df** | **Denominator df** | **F** | **p** |
| --- | --- | --- | --- | --- |
| ***Fixed Factors*** |  |  |  |  |
| Maternal FW age | 2 | 30.62 | 0.12 | 0.886 |
| Paternal FW age | 3 | 45.51 | 1.06 | 0.375 |
| Maternal SW age | 1 | 28.97 | 1.65 | 0.209 |
| Paternal SW age | 3 | 56.66 | 1.934 | 0.134 |
| Stream | 1 | 700.64 | 19.73 | 0.000 |
| Aquarium temperature | 1 | 693.63 | 0.01 | 0.916 |
| ***Covariates*** |  |  |  |  |
| Embryo RTL | 1 | 236.16 | 0.66 | 0.418 |
| Fry density | 1 | 696.59 | 7.74 | 0.006 |
| Predator density | 1 | 703.68 | 1.14 | 0.285 |
| Egg weight | 1 | 35.73 | 11.78 | 0.002 |
| ***Interactions*** |  |  |  |  |
| Maternal SW age x egg weight | 1 | 28.80 | 1.91 | 0.178 |
| Stream x egg weight | 1 | 699.65 | 1.54 | 0.214 |
| Stream x fry density | 1 | 695.71 | 0.08 | 0.784 |
| Stream x predator density | 1 | 703.64 | 1.06 | 0.303 |

**Table S2. Summary of the initial full linear mixed-effect model explaining variation in fry weight (g).** Terms were then sequentially removed if not significant or if not contributing to significant interactions. Mother ID and Father ID were included as random effects to control for non-independence of siblings. See Table 1 for summary of the four final models.

|  | **Numerator df** | **Denominator df** | **F** | **p** |
| --- | --- | --- | --- | --- |
| ***Fixed Factors*** |  |  |  |  |
| Maternal FW age | 2 | 21.65 | 3.07 | 0.067 |
| Paternal FW age | 3 | 31.18 | 0.64 | 0.595 |
| Maternal SW age | 1 | 15.99 | 0.94 | .0347 |
| Paternal SW age | 3 | 29.72 | 0.32 | 0.809 |
| Stream | 1 | 85.81 | 1.13 | 0.290 |
| Aquarium temperature | 1 | 85.89 | 1.21 | 0.275 |
| ***Covariates*** |  |  |  |  |
| Embryo RTL | 1 | 108.87 | 1.35 | 0.248 |
| Fry density | 1 | 93.76 | 0.69 | 0.409 |
| Predator density | 1 | 92.20 | 3.04 | 0.084 |
| Egg weight | 1 | 18.09 | 1.68 | 0.211 |
| ***Interactions*** |  |  |  |  |
| Maternal FW age x stream | 2 | 81.19 | 0.03 | 0.969 |
| Maternal SW age x stream | 1 | 80.76 | <0.01 | 0.987 |
| Paternal FW age x stream | 3 | 80.75 | 0.15 | 0.931 |
| Paternal SW age x stream | 3 | 80.91 | 0.08 | 0.971 |

**Table S3. Summary of the initial full linear mixed-effect model explaining variation in fry survival.** Terms were then sequentially removed if not significant or if not contributing to significant interactions. Mother ID and Father ID were included as random effects to control for non-independence of siblings. See Table 1 for summary of the four final models.

|  | **Numerator df** | **Denominator df** | **F** | **p** |
| --- | --- | --- | --- | --- |
| ***Fixed Factors*** |  |  |  |  |
| Maternal FW age | 2 | 29.14 | 2.66 | 0.087 |
| Paternal FW age | 3 | 44.68 | 0.37 | 0.777 |
| Maternal SW age | 1 | 38.49 | <0.01 | 0.965 |
| Paternal SW age | 3 | 45.48 | 0.89 | 0.455 |
| Time point | 1 | 95.76 | 21.74 | <0.001 |
| Aquarium temperature | 1 | 101.84 | 0.309 | 0.579 |
| ***Covariates*** |  |  |  |  |
| Maternal RTL | 1 | 40.11 | 0.851 | 0.362 |
| Paternal RTL | 1 | 38.62 | 1.571 | 0.218 |
| Egg weight | 1 | 31.44 | 0.483 | 0.492 |

**Table S4. Summary of the initial full linear mixed-effect model explaining variation in embryo telomere length.** Terms were then sequentially removed if not significant. Mother ID and Father ID were included as random effects to control for non-independence of siblings. See Table 1 for summary of the four final models.

|  | **Numerator df** | **Denominator df** | **F** | **p** |
| --- | --- | --- | --- | --- |
| ***Fixed Factors*** |  |  |  |  |
| Maternal FW age | 2 | 25.35 | 2.45 | 0.106 |
| Paternal FW age | 3 | 49.29 | 0.18 | 0.910 |
| Maternal SW age | 1 | 47.04 | 1.55 | 0.219 |
| Paternal SW age | 3 | 64.59 | 2.83 | 0.045 |
| Stream | 1 | 705.41 | 0.95 | 0.331 |
| Aquarium temperature | 1 | 665.38 | 3.20 | 0.074 |
| ***Covariates*** |  |  |  |  |
| Maternal RTL | 1 | 27.01 | 0.45 | 0.507 |
| Paternal RTL | 1 | 35.55 | 0.02 | 0.902 |
| Embryo RTL | 1 | 200.57 | 3.72 | 0.055 |
| Fry weight | 1 | 708.65 | 0.89 | 0.347 |
| Fry density | 1 | 705.13 | 9.58 | 0.002 |
| Predator density | 1 | 708.34 | 6.778 | 0.009 |
| Egg weight | 1 | 34.44 | 2.76 | 0.106 |
| ***Interactions*** |  |  |  |  |
| Fry weight x stream | 1 | 708.96 | 7.17 | 0.008 |
| Fry weight x fry density | 1 | 704.03 | 3.95 | 0.047 |
| Fry weight x predator density | 1 | 708.98 | 2.36 | 0.125 |

**Table S5. Summary of the initial full linear mixed-effect model explaining variation in fry telomere length.** Terms were then sequentially removed if not significant or if not contributing to significant interactions. Mother ID and Father ID were included as random effects to control for non-independence of siblings. See Table 1 for summary of the four final models.

**SUPPORTING RESULTS**

**
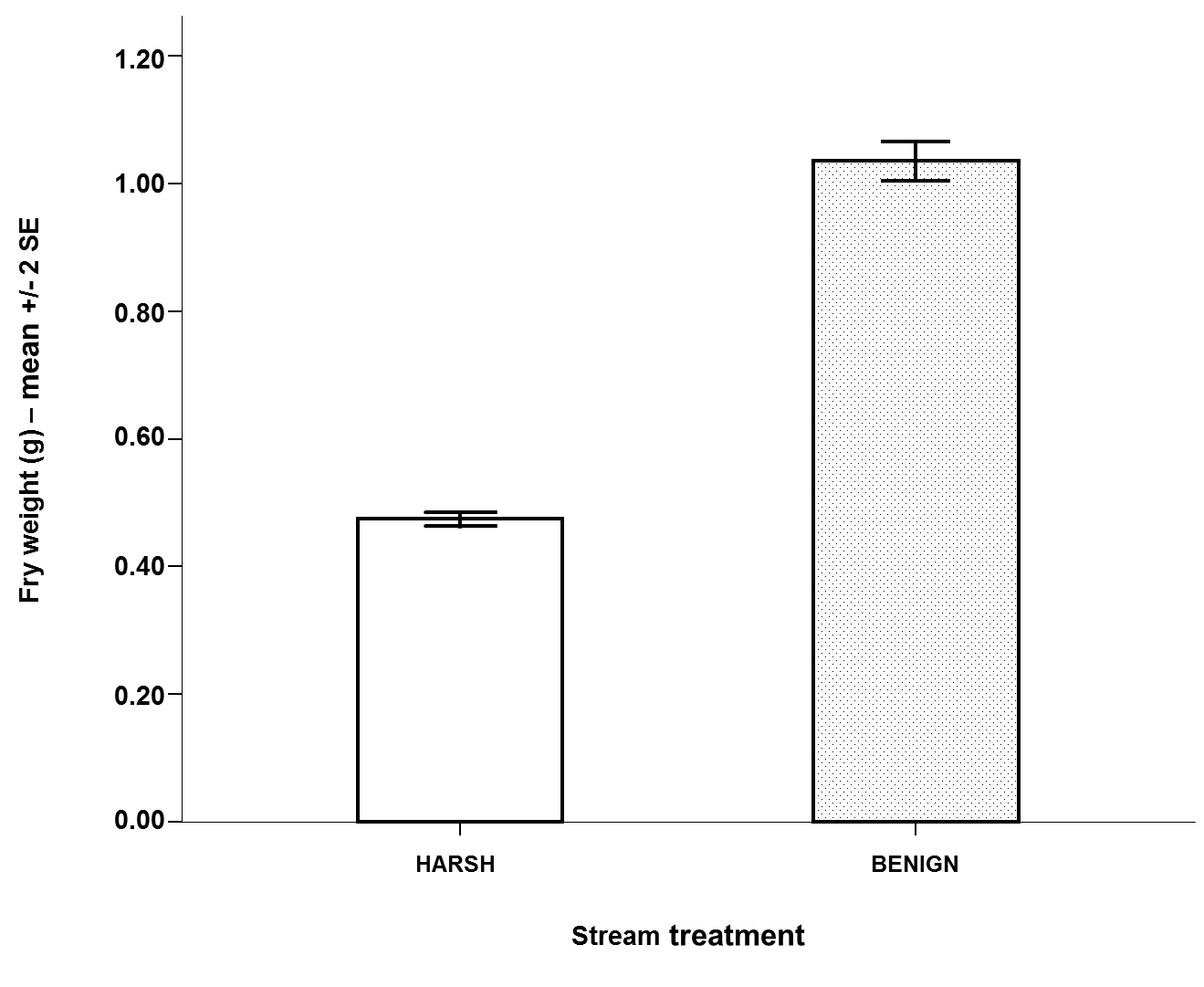
**

**Figure S4. Comparison of the mean fry weight (g) in the two streams at the time of recapture, approximately two months after first feeding.**

**
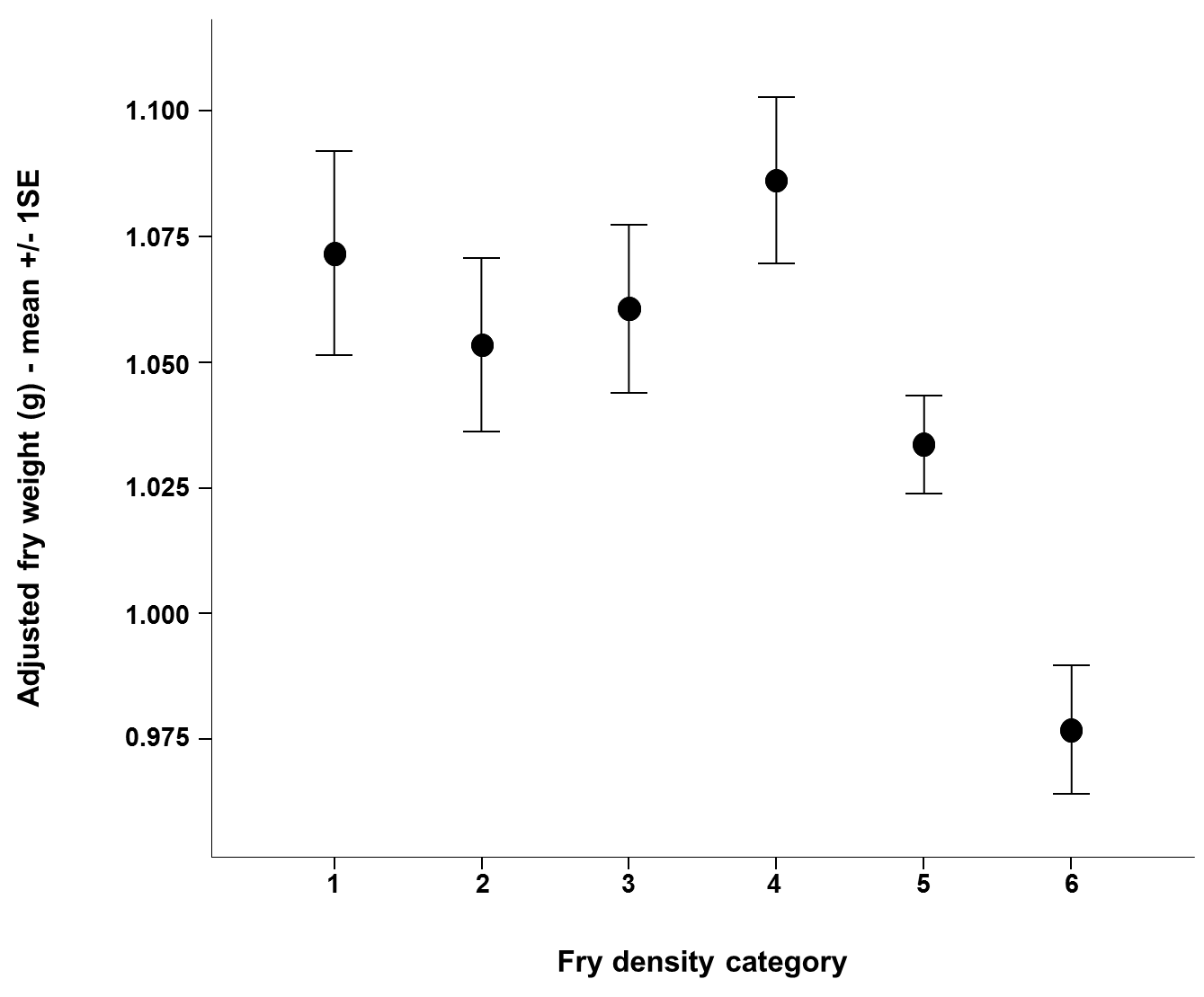
**

**Figure S5. The relationship between fry density and fry weight (g).** Fry weight has been plotted as partial residuals as a function of fry density, evaluated when all other independent variables = 0. Definition of density groups (fry/m^2^): group 1 = <0.2, group 2 =0.21-0.40, group 3 = 0.41-0.60, group 4 = 0.61-0.80, group 5 = 0.81-1.00 and group 6 = >1.01.


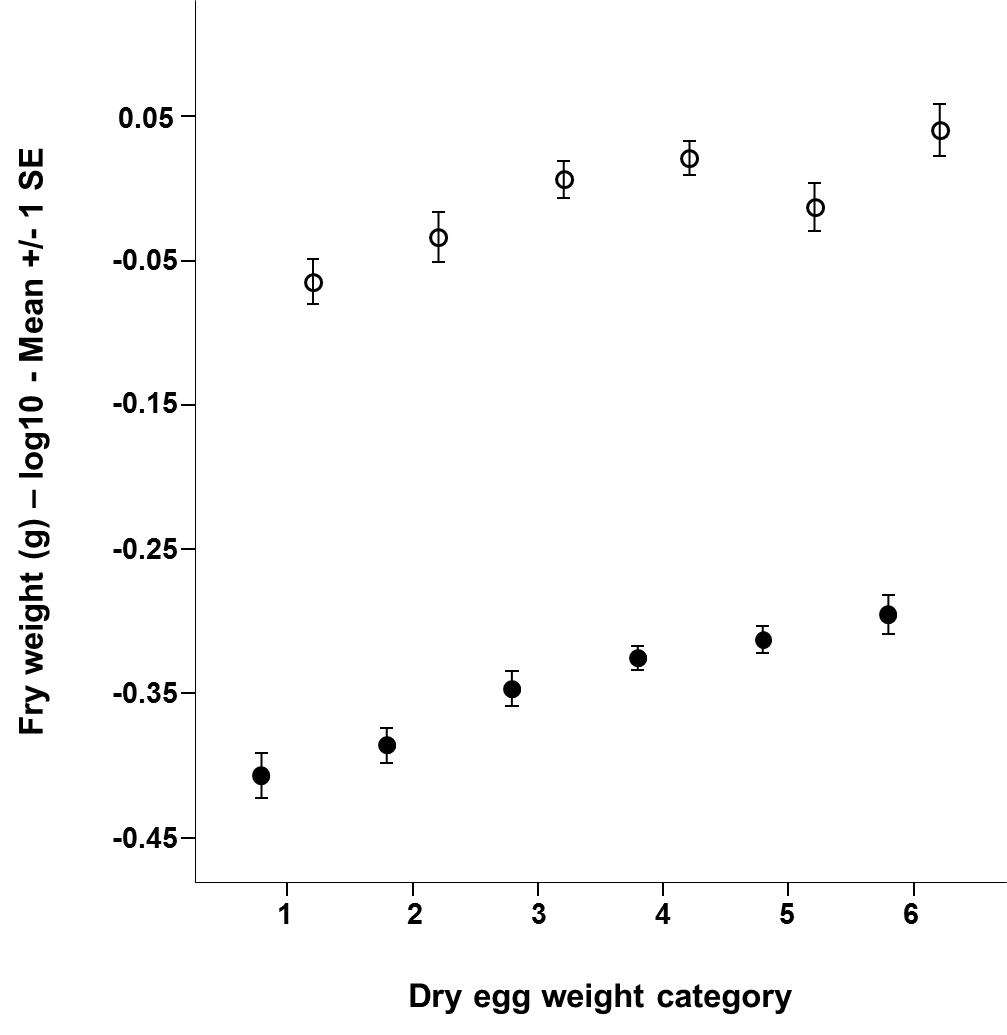


**Figure S6. The relationship between average dry egg weight per family (g) and subsequent fry weight (g) at the time of recapture.** Closed circles = harsh stream, open circles = benign stream. Definition of dry egg weight categories (g): group 1 = 0.030-0.035. group 2 = 0.036-0.040, group 3 = 0.041-0.045, group 4 = 0.046-0.050, group 5 = 0.051-0.055, group 6 = 0.056-0.060.

5
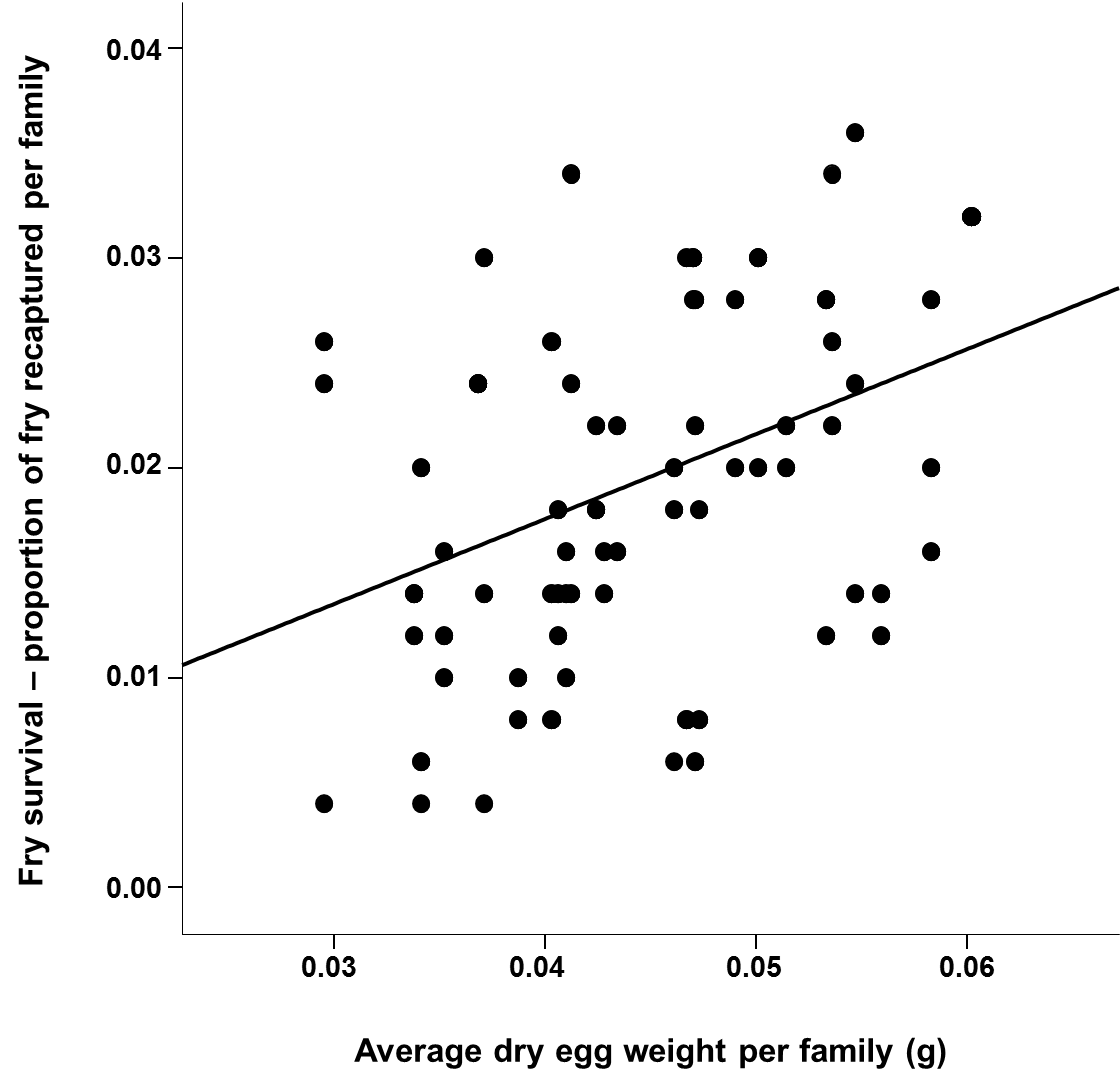


**Figure S7. The relationship between average dry egg weight per family (g) and subsequent fry survival.** Fry survival = proportion of fry recaptured in comparison to the initial number of eggs planted in the stream.


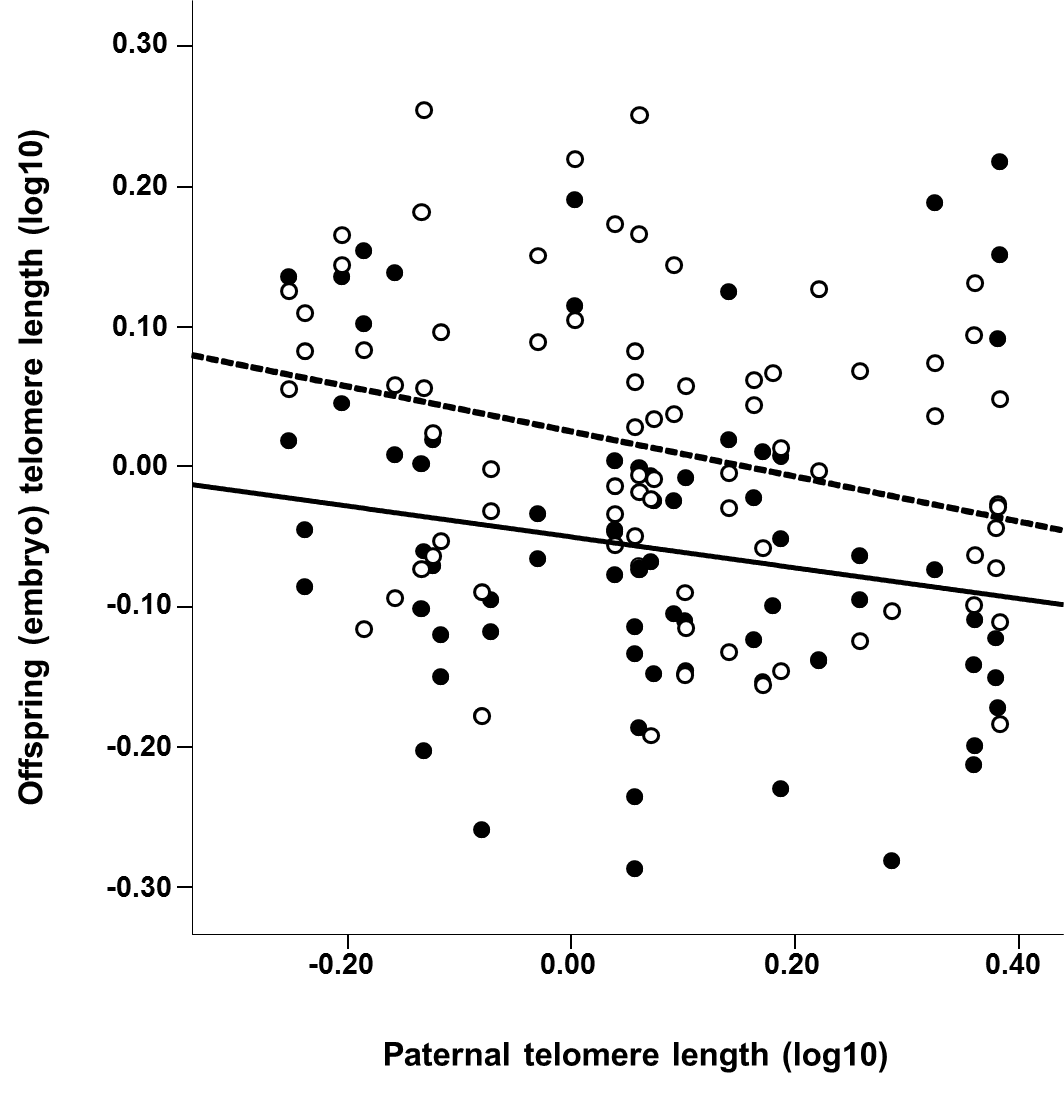


**Figure S8. The relationship between paternal telomere length and embryo telomere length.** Solid circle and solid line = embryo telomere length prior to aquarium temperature treatment. Open circle and dashed line = embryo telomere length after aquarium temperature treatment.

**References**

Aas Ø, Klemetsen A, Einum S, Skurdal J (2011) *Atlantic salmon ecology* Wiley Online Library.

Jonsson B, Jonsson N (2011) *Ecology of Atlantic salmon and Brown trout: habitat as a template for life histories*, 1st edn. Springer, Dordrecht, Netherlands.

Vandeputte M, Mauger S, Dupont-Nivet M (2006) An evaluation of allowing for mismatches as a way to manage genotyping errors in parentage assignment by exclusion. *Molecular Ecology Notes* **6**, 265-267.
